# Transcriptome analysis and functional validation reveal a novel gene, *BcCGF1*, that enhances fungal virulence by promoting infection‐related development and host penetration

**DOI:** 10.1111/mpp.12934

**Published:** 2020-04-16

**Authors:** Ming‐Zhe Zhang, Chen‐Hao Sun, Yue Liu, Hui‐Qiang Feng, Hao‐Wu Chang, Sheng‐Nan Cao, Gui‐Hua Li, Song Yang, Jie Hou, Keyan Zhu‐Salzman, Hao Zhang, Qing‐Ming Qin

**Affiliations:** ^1^ College of Plant Sciences Key Laboratory of Zoonosis Research Ministry of Education Jilin University Changchun, Jilin China; ^2^ College of Plant Sciences Jilin University Changchun, Jilin China; ^3^ College of Computer Science, Technology, Symbol Computation and Knowledge Engineering Ministry of Education Jilin University Changchun, Jilin China; ^4^ College of Forestry BeiHua University Jinlin China; ^5^ Department of Entomology Norman Borlaug Center Texas A&M University College Station TX USA

**Keywords:** *BcCGF1*, *Botrytis cinerea*, conidial germination, differentially expressed genes (DEGs), infection‐related development, mixed transcriptome, pathogenesis, virulence‐associated genes

## Abstract

Simultaneous transcriptome analyses of both host plants and pathogens, and functional validation of the identified differentially expressed genes (DEGs) allow us to better understand the mechanisms underlying their interactions. Here, we analyse the mixed transcriptome derived from *Botrytis cinerea* (the causal agent of grey mould) infected tomato leaves at 24 hr after inoculation, a critical time point at which the pathogen has penetrated and developed in the leaf epidermis, whereas necrotic symptoms have not yet appeared. Our analyses identified a complex network of genes involved in the tomato–*B. cinerea* interaction. The expression of fungal transcripts encoding candidate effectors, enzymes for secondary metabolite biosynthesis, hormone and reactive oxygen species (ROS) production, and autophagy‐related proteins was up‐regulated, suggesting that these genes may be involved in the initial infection processes. Specifically, tomato genes involved in phytoalexin production, stress responses, ATP‐binding cassette transporters, pathogenesis‐related proteins, and WRKY DNA‐binding transcription factors were up‐regulated. We functionally investigated several *B. cinerea* DEGs via gene replacement and pathogenicity assays, and demonstrated that *BcCGF1* was a novel virulence‐associated factor that mediates fungal development and virulence via regulation of conidial germination, conidiation, infection structure formation, host penetration, and stress adaptation. The fungal infection‐related development was controlled by *BcCGF*‐mediated ROS production and exogenous cAMP restored the mutant infection‐related development. Our findings provide new insights into the elucidation of the simultaneous tactics of pathogen attack and host defence. Our systematic elucidation of *BcCGF1* in mediating fungal pathogenesis may open up new targets for fungal disease control.

## Graphical abstract

Transcriptome profiling of both tomato and grey mould fungus at an early stage of infection and functional analysis reveal a novel pathogen factor, BcCgf1, that promotes fungal virulence by mediating infection‐related development.

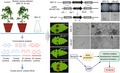

## 1 INTRODUCTION

The necrotrophic fungal pathogen *Botrytis cinerea* causes grey mould on over 1,000 plant species (Fillinger and Elad, 2016). The pathogen infects almost all vegetable and fruit crops, including numerous economically important crops such as grapevine, strawberry, and raspberry, and annually causes enormous economic losses worldwide. The pathogen attacks many plant organs, including stems, fruits, leaves, and flowers, at both pre‐ and post‐harvest stages (Dean *et al.*, 2012). In the field, the main infection source of this pathogen is conidia that form appressoria or appressorium‐like structures on host surfaces to facilitate infection (Gourgues *et al.*, 2004; Choquer *et al.*, 2007). In addition, *B. cinerea* is the most extensively studied necrotrophic fungal pathogen due to the availability of its genome sequence, its ease of study (e.g., easy to obtain gene knockout mutants or achieve gene silencing), as well as its economic relevance (Dean *et al.*, 2012).

*B. cinerea* employs a range of toxic molecules to kill and decompose plant tissue prior to converting it into fungal biomass (Williamson *et al.*, 2007). To initiate host infection, *B. cinerea* conidia on plant surfaces germinate and form appressoria for host penetration. Conidia of the pathogen hardly germinate in the presence of only water. Therefore, gluconeogenesis and glucose play a crucial role in the initiation of conidial germination; gluconeogenesis allows the pathogen to cope with the limitation of glucose and/or other carbon sources in the infection niches (Liu *et al.*, 2018). Shortly after conidial germination, the germ tubes cease their polarized cell growth and start to form swollen melanized structures called appressoria. Besides appressoria, *B. cinerea* also forms highly melanized specialized hyphal networks or clumps of hyphal structures called infection cushions (Marschall and Tudzynski, 2016b). Infection structures (IFSs), including appressoria and infection cushions, are required for the pathogen to penetrate host cells. The fungal septin protein Sep4 is essential for pathogens to initiate IFS formation and host penetration (Feng *et al.*, 2017). Plant cell walls are barriers that impair pathogen host colonization; however, they are also important reservoirs of energy‐rich sugars. To invade plant cells and to degrade or exploit cell wall components, *B. cinerea* secretes enzymes that disassemble cell wall polysaccharides during infection (Blanco‐Ulate *et al.*, 2014). Interestingly, to disarm host resistance, the fungus also employs small RNAs as effectors that are delivered to the host plant cells where they can be loaded by the RNA silencing machinery and target plant defence genes (Weiberg *et al.*, 2013). Although knowledge about *B. cinerea* pathogenesis has greatly expanded in the last two decades, mechanisms of pathogen factors that mediate the fungal pathogenesis and/or disarm host resistance remain obscure.

Once a pathogen is detected, the host plant activates signalling networks through the generation of small signalling molecules and coordination of hormonal signalling pathways to initiate defence mechanisms to the pathogen attack (AbuQamar *et al.*, 2017). Plant responses to *B. cinerea* infection include host cell death, production of various secondary metabolites, antimicrobial peptides, and hormones, for example ethylene (ET), salicylic acid (SA), abscisic acid (ABA), and jasmonate (JA), as well as accumulation of reactive oxygen species (ROS), callose, and a variety of other cell wall modifications (Mengiste, 2012). ABA, ET, and auxin, are known to be actively involved in plant defence against *B. cinerea* (Navarro *et al.*, 2006; El Oirdi *et al.*, 2011; Windram *et al.*, 2012; Sivakumaran *et al.*, 2016). Other phytohormones, for example brassinosteroids, also regulate plant immunity by mainly interacting with transcription factors, or through camalexin biosynthesis and callose deposition. In addition, studies have notably provided evidence for cross‐talk among SA, JA, and ET or with other hormones in regulating plant defence responses to *B. cinerea* (for review, see AbuQamar *et al.*, 2017).

Long non‐coding RNAs (lncRNAs) play important roles in gene expression and silencing pathways for several biological processes in eukaryotes. Host lncRNAs also play roles in plant defence. In response to pathogen infection, lncRNA‐dependent immune systems protect host plants via lncRNAs’ regulation of pathogen‐associated molecular patterns (PAMPs) and other effectors (Zaynab *et al.*, 2018). *Arabidopsis* cells secrete exosome‐like extracellular vesicles to deliver small RNAs (sRNAs) into *B. cinerea* to silence fungal genes critical for pathogenicity, indicating that *Arabidopsis* has employed exosome‐mediated cross‐kingdom RNA interference as part of its immune responses during the evolutionary arms race with the pathogen (Cai *et al.*, 2018). Despite some understanding of the antifungal mechanisms, more extensive studies on plant resistance to the pathogen are necessary, especially on host plants that are internally colonized by aggressive *B. cinerea* strains without displaying any signs of disease or stress (Shaw *et al*., 2016; Veloso and van Kan, 2018).

In *B. cinerea* pathosystems, host plant and/or pathogen transcriptomes have been investigated in *Arabidopsis* (Mulema and Denby, 2012), tomato (Blanco‐Ulate *et al.*, 2013; Smith *et al.*, 2014; Vega *et al.*, 2015; Rezzonico *et al.*, 2017), lettuce (De Cremer *et al.*, 2013), and cucumber (Kong *et al.*, 2015). These reports greatly increase our understanding of the mechanisms underlying *B. cinerea* interaction with its diverse hosts. However, plant or pathogen factors identified in the transcriptome analysis during their interactions remain to be functionally validated.

In this study, we investigated the simultaneous gene expression profiles of *B*. *cinerea* and tomato and identified a complex network of differentially expressed genes (DEGs) involved in the early stage of the *B*. *cinerea–*tomato interaction. We also functionally investigated several up‐regulated fungal genes and demonstrated that *BcCGF1* is a novel *B. cinerea* factor that mediates the process of fungal development and infection via facilitating ROS production. Our work provides new insights into the molecular mechanisms underlying tomato–*B*. *cinerea* interaction at the early stage, which may be crucial to protect plants from damage caused by necrotrophic fungal pathogens.

## 2 RESULTS

### 2.1 DEGs in tomato and *B. cinerea* at the early interacting stage (24 hr post‐inoculation)

Transcriptomic analyses of *B. cinerea*‐infected tomato leaves as well as plant and pathogen controls (uninfected tomato plants and *B. cinerea* B05.10 cultured in ½ × potato dextrose broth, PDB) generated a total of 83,128,534 raw reads. After removing the adapter sequences and low‐quality reads, 70,186,664 clean reads remained (Table S1). From these, 96.2% and 84.4% of the clean reads in control and infected tomato, respectively, were mapped to the reference tomato genome. Likewise, 95.2% and 6.1% of the clean reads in *B. cinerea* at the noninfection and infection stages, respectively, were mapped to the *B. cinerea* genome (Tables S1 and S2). In tomato plants, RNA‐Seq yielded 1,319 DEGs (|log_2_FC [fold change]|> 1, *p* < .01); among them, 720 were up‐regulated and 599 were down‐regulated (Figure S1a, and Tables S3 and S4). In the fungus, 918 DEGs were detected and 621 and 297 DEGs were up‐regulated and down‐regulated, respectively (Figure S1b, and Tables S5 and S6). Our gene ontology (GO) and Kyoto Encyclopedia of Genes and Genomes (KEGG) enrichment analyses of tomato and *B. cinerea* DEGs revealed that the enriched GO terms (Figure S1c,d, and Tables S7 and S8) and KEGG pathways (Figure S2, and Tables S9 and S10) of the DEGs were similar to those previously reported (De Cremer *et al.*, 2013; Kong *et al.*, 2015; Rezzonico *et al.*, 2017).

### 2.2 Characteristics of up‐regulated genes in *B. cinerea‐*infected tomato leaves

To understand early plant responses to *B. cinerea* infection, we selected some up‐regulated tomato genes for further functional annotation (Tables 1 and S3). Presumably, these transcripts play important roles in tomato plants against *B. cinerea* infection.

**TABLE 1:** Highly up‐regulated (fold change > 50) tomato genes

Gene ID	Protein symbol	Fold change	Annotation
Solyc03g020050.2	CEVI57	1911.6	Proteinase inhibitor type‐2 CEVI57
Solyc06g066230.2	LOC101248402	476.2	Cytochrome P450 71D7
Solyc02g078650.2	LOC101263372	460.1	Polyphenol oxidase, chloroplastic
Solyc12g010980.1	LOC101260610	389.8	Salutaridinol 7‐*O*‐acetyltransferase
Solyc04g072280.2	LOC101261512	300.2	Laccase‐14
Solyc12g088800.1	LOC101263295	273.1	Hypothetical protein
Solyc01g101210.2	TPS35	244.6	Vetispiradiene synthase 1
Solyc08g007090.1	Solyc08g007090.1.1	226.2	Expansin‐like B1
Solyc05g021390.2	LOC101251676	223.9	Cytochrome P450 716B2
Solyc01g101190.2	TPS33	214.8	Vetispiradiene synthase 1
Solyc08g006750.2	LOC101249664	178.5	Histidine decarboxylase
Solyc00g247300.2	LOC101247602	166.7	Cytochrome P450 84A1
Solyc01g095080.2	ACS2	161.1	1‐aminocyclopropane‐1‐carboxylate synthase 2
Solyc02g080840.1	LOC101256060	157.3	Probable F‐box protein At4g22030
Solyc09g066400.1	LOC101248046	156.6	Premnaspirodiene oxygenase
Solyc02g070110.1	LOC101263972	136.4	Cannabidiolic acid synthase
Solyc04g016000.2	LOC101266325	136.4	Heat stress transcription factor B‐3
Solyc01g101170.2	TPS31	133.2	Probable 5‐epi‐aristolochene synthase 4
Solyc07g062370.1	LOC101249107	122.5	Hypothetical protein
Solyc02g067750.2	CA3	115.5	Carbonic anhydrase, chloroplastic
Solyc01g101180.2	TPS32	109.5	Viridiflorene synthase
Solyc03g119370.1	LOC101266622	107.5	Myb‐related protein 305
Solyc08g036660.2	LOC101253212	105.8	Hypothetical protein
Solyc08g006740.2	AADC2	100.0	Histidine decarboxylase
Solyc02g070090.1	LOC101255854	92.9	Reticuline oxidase‐like protein
Solyc02g038740.2	HMG2	88.0	3‐hydroxy‐3‐methylglutaryl‐coenzyme A reductase 2
Solyc01g087280.1	LOC101263946	88.0	Polygalacturonase
Solyc02g088160.2	LOC101267864	87.3	Casparian strip membrane protein
Solyc05g046340.1	LOC101264724	84.0	Phosphomannomutase
Solyc02g078930.1	LOC101268840	82.6	Hypothetical protein
Solyc09g075700.1	LOC101255920	79.7	Probable carboxylesterase 13
Solyc04g083140.1	LOC101251277	78.9	Premnaspirodiene oxygenase
Solyc04g071070.2	LOC544158	73.4	Hypothetical protein
Solyc11g069560.1	LOC101262484	71.4	Hypothetical protein
Solyc07g064600.2	CHRDi	66.8	RutC family protein C23G10.2
Solyc06g065060.1	LOC101254908	64.9	Cannabidiolic acid synthase
Solyc09g013150.2	LOC101254229	63.0	Probable anion transporter 3, chloroplastic
Solyc11g067000.1	ABCG51	62.7	Pleiotropic drug resistance protein 2
Solyc07g005380.2	LOC101262431	61.7	S‐norcoclaurine synthase
Solyc01g107080.2	LOC101265373	61.5	Uncharacterized acetyltransferase At3g50280
Solyc09g011520.2	LOC101267638	60.4	Probable glutathione S‐transferase
Solyc01g005390.2	LOC101248702	59.2	Nudix hydrolase 18, mitochondrial
Solyc03g005500.1	LOC101261887	58.7	Ethylene‐responsive transcription factor ERF098
Solyc02g093180.2	LOC101266883	58.2	Uncharacterized acetyltransferase At3g50280
Solyc06g007180.2	AS1	56.7	Asparagine synthetase
Solyc05g047530.2	LOC101262919	56.6	Trans‐cinnamate 4‐monooxygenase
Solyc12g006530.1	lTTS1	56.2	β‐amyrin synthase
Solyc04g074770.2	LOC101267111	54.6	Unknown protein
Solyc10g084240.1	LOC101249874	54.6	Peroxidase 21
Solyc07g054720.1	LOC101256195	52.3	Proteinase inhibitor type‐2 CEVI57
Solyc05g056170.2	PAL2	51.8	Phenylalanine ammonia‐lyase

#### 2.2.1 Genes related to ROS and phytohormone production

In response to *B. cinerea* infection, several up‐regulated genes encoding ROS‐generating enzymes, including *Solyc02g087070.2* (*LOC543895*), *Solyc02g079500.2* (*TMP1*), *Solyc02g078650.2* (*LOC101263372*), *Solyc07g043590.2* (*LOC101252005*), and *Solyc10g084240.1* (*LOC101249874*), were identified (Tables 1 and S3). The two type I 1‐amino‐cyclopropane‐1‐carboxylic acid synthase (ACS) isozymes ACS2 and ACS6 are responsible for *B. cinerea*‐induced ET production (Han *et al.*, 2010). Many genes involved in ET biosynthesis were up‐regulated, including genes related to ET response factor (ERF) transcription factors (*Solyc03g005500.1* [*LOC101261887*], *Solyc05g051200.1* [*LOC606712*], *Solyc06g053710.2* [*ETR4*], *Solyc08g078180.1* [*ERF‐A1*], *Solyc09g066360.1* [*ERF‐C3*], *Solyc12g056590.1* [*ERF‐D2*], *Solyc01g095080.2* [*ACS2*], *Solyc03g080190.2* [*DMR6‐1*], and *Solyc12g005940.1* [*ACO2*]) (Tables 1 and S3). Genes involved in biosynthetic pathways of ABA, for example *Solyc04g071590.1* (*ASR3*) and *Solyc04g071600.2* (*LOC101247814*), and auxin, for example *Solyc03g120390.2* (*IAA15*) and *Solyc03g121060.2* (*IAA26*), were also up‐regulated (Table S3).

#### 2.2.2 Genes related to ATP‐binding cassette transporters

**FIGURE 1:**
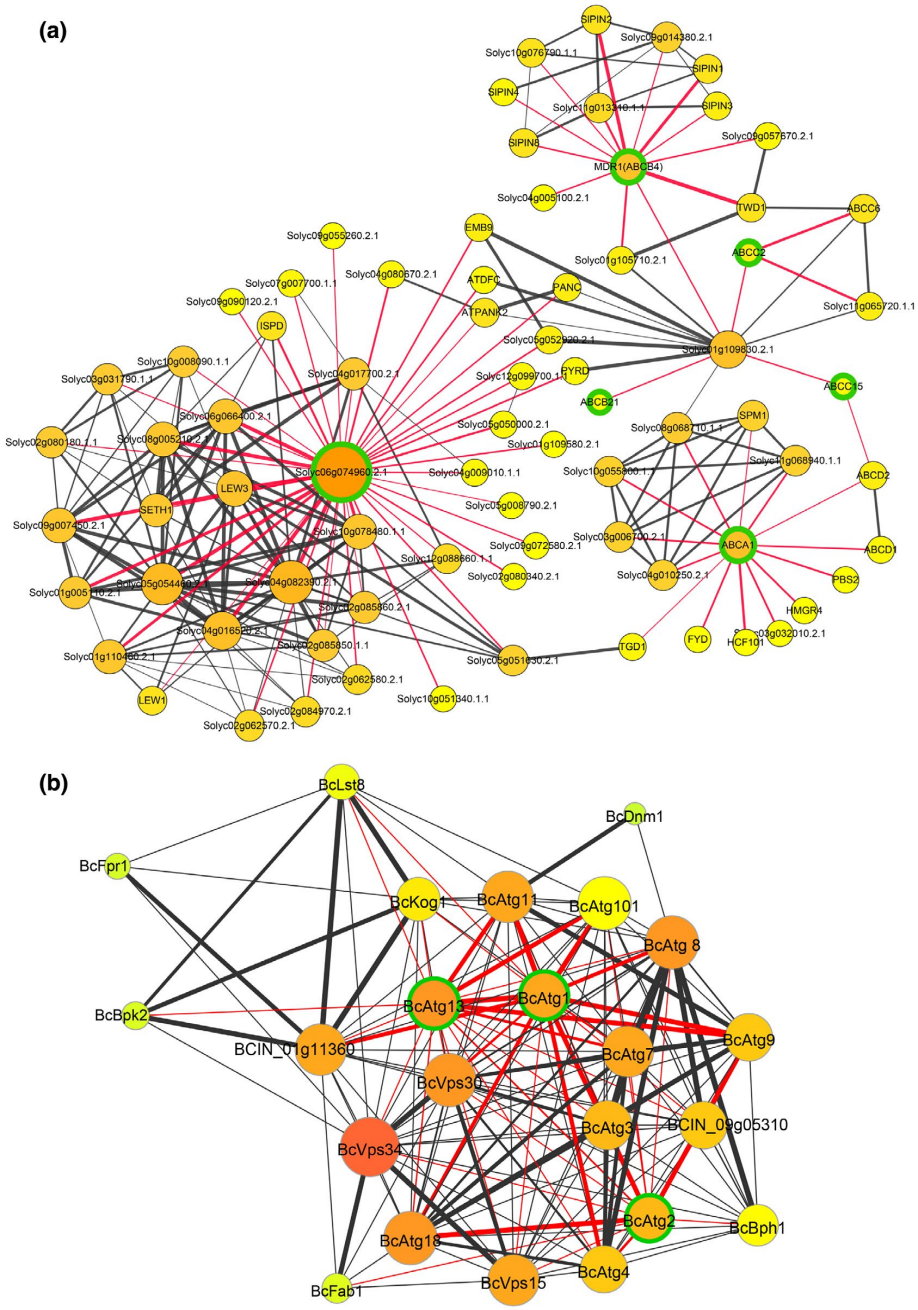
The protein–protein interaction (PPI) networks of tomato and *Botrytis cinerea* in the early stage of their interaction. The PPI networks of tomato ATP‐binding cassette (ABC) transporters (a) and *B. cinerea* autophagy‐related proteins (b) in the early stage of tomato–*B. cinerea* interaction. The proteins with green edges are encoded by the corresponding up‐regulated tomato ABC transporter genes (a) or *B. cinerea* autophagy‐related genes (b). The other interaction proteins in the networks are from STRING (https://string-db.org/), Uniprot (https://www.uniprot.org/), and NCBI (https://www.ncbi.nlm.nih.gov/). For the size of the circles, the bigger the circles, the more connecting strings/lines or interacting proteins the circles have. For the filled colours in the circles, the more connecting lines, the closer the colour to orange (ranging from yellow to orange). For the colours of the connecting lines, red indicates that proteins directly interact with the interested proteins (nodes with green edges) and black indicates other relationships of interactions. The bold connecting lines indicate that the combined score (0–1, the higher the score, the more reliable the interaction relationship) is greater than 0.95

ATP‐binding cassette (ABC) transporters are involved in improving plant immunity (Mulema and Denby, 2012). Genes related to ABC transporters, including *Solyc00g233480.1*, *Solyc02g087870.2* (*MDR1*), *Solyc03g007530.2* (*ABCC2*), *Solyc04g015970.2* (*ABCA1*), *Solyc05g014380.2* (*LOC101257768*), *Solyc05g014390.2*, *Solyc06g009290.2* (*ABCB9*), and *Solyc06g074960.2* (*LOC101266730*), were up‐regulated in the infected leaves (Tables 1 and S3). Proteins encoded by these genes are classified in diverse ABC transporter families (Table S11). Our protein–protein interaction (PPI) network analysis suggested that the ABC transporters encoded by these genes interact with many other ABC transporters or related proteins (Figure 1a, Table S11) in response to *B. cinerea* infection.

#### 2.2.3 Genes associated with autophagy machinery and WRKY DNA‐binding transcription factors

Autophagy is a mechanism of the cell that disassembles and recycles unnecessary or dysfunctional cellular components. The up‐regulation of an autophagy‐related (ATG) gene *SlATG8* (*Solyc08g078820.2*) (Table S3) suggests that autophagy machinery plays a role in the tomato response to *B. cinerea* infection and may be conducive to host cell survival, in agreement with previous reports (Lai *et al.*, 2011; Windram *et al.*, 2012). Tomato WRKY DNA‐binding transcription factors mediate disease resistance against *B. cinerea* (Liu *et al.*, 2014). Three tomato genes *Solyc02g080890.2* (*SlWRKY6*), *Solyc02g094270.1*, and *Solyc05g015850.2* (*SlWRKY75*) encoding WRKY DNA‐binding transcription factors were up‐regulated in response to *B. cinerea* attack (Table S3).

#### 2.2.4 Genes related to PAMP receptors, PR proteins, and other factors involved in host defence

Fungal cell wall components such as oligosaccharides and chitin are fungal PAMPs that are recognized by host plant receptors and activate defence responses. Fungal polygalacturonases (PGs) are important components essential for virulence and detected by host plants (Windram *et al.*, 2012). The tomato chitin elicitor receptor kinase gene *Solyc04g072000.2* (*LOC101256086*) that encodes chitinase (Liu *et al.*, 2012), and genes *Solyc02g070910.1* (*LOC101259509*) and *Solyc07g006770.2* (*LOC101243723*) encoding flagellin‐sensitive 2 (FLS2) that improve immune responses in *Arabidopsis* (Sun *et al.*, 2013) were up‐regulated (Table S3). *Arabidopsis* Kazal‐type serine proteinase inhibitors (KPIs) are induced in response to *B. cinerea* infection and AtKPI‐1 displays a strong antifungal activity in inhibition of the pathogen conidial germination, demonstrating the roles of KPIs in defence against pathogens (Pariani *et al.*, 2016). During *B. cinerea* infection, tomato genes related to KPI (*LOC101256195*), pathogenesis‐related (PR) proteins PR1b1 (*Solyc00g174340.1*), PR‐1a1 (*Solyc01g106610.2*), and *Solyc01g097240.2* (PR‐P2), wounding *Solyc01g097270.2* (*LOC543758*), peroxidases, for example *Solyc10g084240.1* (Peroxidase 21), *Solyc02g094180.2*, *Solyc01g105070.2*, and *Solyc01g007950.2* (Peroxidase 1), and respiratory burst *Solyc01g099620.2* (*LOC101251928*) were up‐regulated (Table S3).

### 2.3 Characteristics of up‐regulated genes in *B. cinerea* at early stage of infection

To understand the possible roles of pathogen genes in host infection, we characterized some up‐regulated fungal DEGs, including *BCIN_12g01020* encoding oxaloacetate acetylhydrolase (OAH) and *BCIN_03g01540* encoding choline dehydrogenase (Tables 2 and S5). The pathogen may orchestrate these DEGs to facilitate host infection.

**TABLE 2:** Highly up‐regulated (fold change ≥ 40) *Botrytis cinerea* genes

Gene ID	Protein symbol	Fold change	Annotation	Secreted
BCIN_03g01540	BCIN_03g01540	527.7	Choline dehydrogenase	Yes
BCIN_12g01020	Bcoah	402.4	Oxaloacetate acetylhydrolase (Bcoah1)	No
BCIN_15g05630	BCIN_15g05630	349.4	Putative NAD‐dependent epimerase dehydratase family protein	No
BCIN_15g01960	BCIN_15g01960	211.3	RNA methyltransferase, TrmH family, group 3	Yes
BCIN_08g00280	BCIN_08g00280	206.8	Similar to carboxypeptidase S1	Yes
BCIN_06g00650	BCIN_06g00650	202.1	Isotrichodermin C‐15 hydroxylase	No
BCIN_12g06300	BCIN_12g06300	202.0	Similar to metalloproteinase	No
BCIN_01g01260	BCIN_01g01260	171.3	DJ‐1/PfpI family protein DJ‐1	No
BCIN_16g01820	BCIN_16g01820	156.1	Conidial germination protein/ Bccgf1	Yes
BCIN_03g01560	BCIN_03g01560	151.8	Similar to aldo/keto reductase	No
BCIN_02g01260	BCIN_02g01260	134.0	Pisatin demethylase	No
BCIN_03g05820	BCIN_03g05820	106.0	Pectate lyase B precursor	Yes
BCIN_12g00380	BCIN_12g00380	97.2	Hypothetical protein	Yes
BCIN_15g02380	Bcacp1	95.0	Acidic protease	Yes
BCIN_03g01520	BCIN_03g01520	78.0	NADP‐dependent alcohol dehydrogenase	No
BCIN_12g02040	Bcap8	76.3	Polyporopepsin/Bcap8	Yes
BCIN_02g08100	BCIN_02g08100	76.0	Hypothetical protein	No
BCIN_15g03320	BCIN_15g03320	75.6	Acid phosphatase precursor	Yes
BCIN_14g01070	BCIN_14g01070	74.0	Hypothetical protein similar to alcohol dehydrogenase	No
BCIN_05g04180	BCIN_05g04180	73.8	Hypothetical protein similar to betaine lipid synthase	No
BCIN_01g01550	BCIN_01g01550	72.3	Hypothetical protein	No
BCIN_06g00130	BCIN_06g00130	70.0	Oligopeptide transporter	No
BCIN_04g02930	Bmr5	63.6	Macrolide transporter ATP‐binding/permease protein	No
BCIN_02g04840	BCIN_02g04840	62.9	Inorganic phosphate transporter 1‐6/Pi cotransporter	No
BCIN_09g01150	BCIN_09g01150	62.6	Hypothetical protein	Yes
BCIN_03g03630	BCIN_03g03630	61.1	Endoglucanase A precursor	Yes
BCIN_11g02900	BCIN_11g02900	60.0	Trypsin	Yes
BCIN_06g05050	BCIN_06g05050	59.9	Hypothetical protein similar to glucoamylase	Yes
BCIN_02g08710	BCIN_02g08710	58.4	Hypothetical protein	No
BCIN_14g04260	Bcgas2	57.6	Protein of unknown function	Yes
BCIN_10g05620	BCIN_10g05620	55.3	Pectate lyase precursor	Yes
BCIN_05g01510	BCIN_05g01510	51.7	Hypothetical protein	Yes
BCIN_07g04510	BCIN_07g04510	50.9	Hypothetical protein	No
BCIN_09g02930	Bcap1	49.5	Aspergillopepsin A precursor (Bcap1)	No
BCIN_08g04910	BCIN_08g04910	49.5	Hypothetical protein	No
BCIN_04g05960	BCIN_04g05960	49.2	Hypothetical protein	Yes
BCIN_13g03660	BCIN_13g03660	47.9	Hypothetical protein	No
BCIN_14g05330	BCIN_14g05330	44.9	Similar to GPI anchored dioxygenase	Yes
BCIN_07g05430	BCIN_07g05430	42.8	Cytochrome P450 3A10	No
BCIN_05g07630	BCIN_05g07630	41.7	Hypothetical protein	Yes
BCIN_06g00120	BCIN_06g00120	41.3	Oligopeptide transporter 7	No
BCIN_10g01510	BCIN_10g01510	40.9	Hypothetical protein	Yes
BCIN_14g01770	BCIN_14g01770	40.0	Putative MFS multidrug protein	No
BCIN_16g02770	Bcmp1	40.0	Peptidase M35 domain of deuterolysins and related proteins	Yes

#### 2.3.1 Genes involved in secondary metabolite biosynthesis and toxin production

Oxalate secretion by fungal pathogens is usually associated with their pathogenesis (Han *et al.*, 2007; Liang *et al.*, 2015). The VelB/VeA/LaeA complex coordinates light signal with fungal development and secondary metabolism (Bayram *et al.*, 2008; Schumacher *et al.*, 2015). During infection, fungal genes involved in secondary metabolites and toxin production were up‐regulated. For example, *BCIN_12g01020* encoding BcOah1, *BcVEL1* (*BCIN_15g03390*) and *BcLAE1* (*BCIN_05g01210*) involved in secondary metabolism and virulence (Yang *et al.*, 2013; Schumacher *et al.*, 2015), and *BCIN_03g01570* encoding polyketide synthases that regulates phytotoxin botcinic acid biosynthesis (Dalmais *et al.*, 2011) were highly expressed (Tables 2 and S5).

#### 2.3.2 Genes related to hormone and ROS production

Cytochrome P450 monooxygenases are essential for ABA biosynthesis in *B. cinerea* (Siewers *et al.*, 2004). ABA decreases tomato resistance to *B. cinerea* via reduction of NO production, which also suppresses both ROS and ET production (Sivakumaran *et al.*, 2016). Two P450 monooxygenase genes (*BCIN_07g05430* and *BCIN_03g06490*) involved in ABA biosynthesis in *B. cinerea* were up‐regulated (Table S5). NADPH oxidases are required for ROS production and are involved in conidial germination, differentiation, and vegetative and pathogenicity development as well as virulence in *B. cinerea* (Marschall and Tudzynski, 2016a; Cao *et al.*, 2018; Hou *et al*., 2020). NADPH oxidase genes *BcNOXA* (*BCIN_05g00350*) and *BcNOXB* (*BCIN_02g04930*) were also up‐regulated (Table S5).

#### 2.3.3 Autophagy‐related genes

At the initiation of infection, the pathogen ATGs, including *ATG1* (*BCIN_07g00720*), *ATG2* (*BCIN_14g01550*), and *ATG13* (*BCIN_13g04910*), were up‐regulated (Table S5). Our PPI network analysis suggests that the autophagy proteins encoded by these ATGs interact with many proteins, including Atg1, Atg3, Atg4, Atg7, and Atg8 (Figure 1b, Table S12).

#### 2.3.4 Genes encoding carbohydrate‐active enzymes

Growing hyphae of *B. cinerea* secrete a large number of extracellular virulence components including cell wall degradation (CWD)‐associated enzymes that disassemble cell wall polysaccharides. *B. cinerea* polygalacturonases are essential for its virulence and can be detected by host plants (Blanco‐Ulate *et al.*, 2014). Cutinases are extracellular degradative enzymes that hydrolyse cutin and facilitate fungal penetration through the cuticle (van der Vlugt‐Bergmans *et al.*, 1997). During infection, a group of genes encoding enzymes involved in CWD were up‐regulated. These genes included two pectate lyase genes (*BCIN_03g05820* and *BCIN_10g05620*), three endoglucanase genes (*BCIN_10g06130*, *BCIN_12g06630*, and *BCIN_05g07690*), a cutinase‐related gene (*BCIN_03g04560*), one endo‐1,4‐β‐xylanase gene (*BCIN_12g00090*), eight glucosidase‐related genes (e.g. *BCIN_09g02640* and *BCIN_09g05460*), and three PG genes (*BcPG3* [*BCIN_04g04930*], *BcPG4* [*BCIN_03g01680*], and *BcPG6* [*BCIN_02g05860*]) (Table S5).

#### 2.3.5 Genes encoding candidate effectors

*B*. *cinerea* effectors play important roles in establishment of infection via suppression of plant innate immunity (Weiberg *et al.*, 2013; Heard *et al.*, 2015). Through prediction of secretory signal peptide and the cellular localization of protein encoded by each up‐regulated fungal transcript, we identified a larger number of transcripts (88/621) encoding putative secreted proteins that may be *B. cinerea* candidate effectors (Table S13). Among them, several candidate effector genes were highly up‐regulated, for example the expression levels of *BCIN_16g01820* and *BCIN_10g05620*, encoding a hypothetic protein and a pectate lyase, respectively, were over 150‐ and 60‐fold of that of the control (Table 2).

### 2.4 Functional analysis of up‐regulated *B. cinerea* genes

**FIGURE 2:**
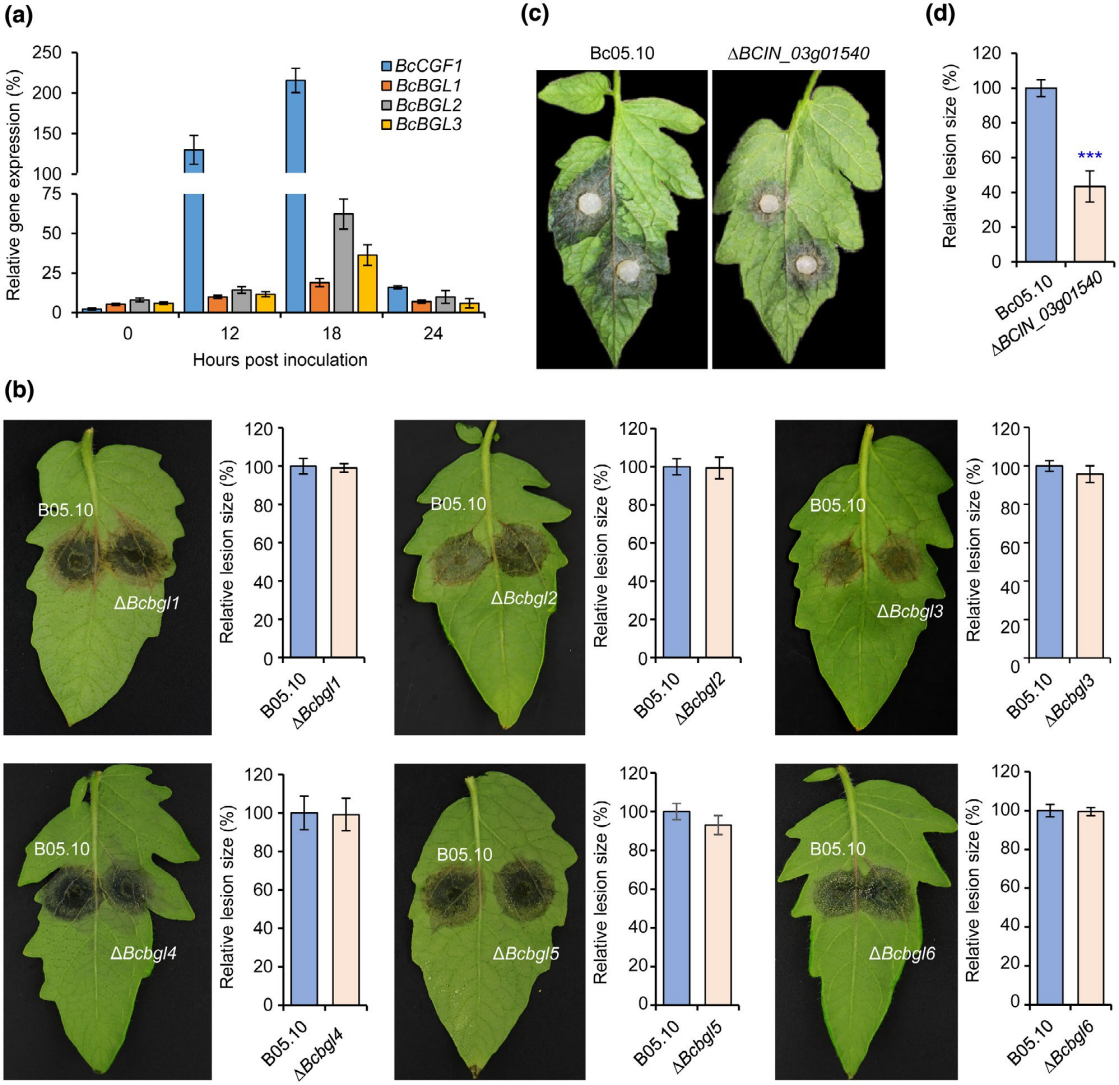
Functional analysis of the differentially expressed genes in *Botrytis cinerea*. (a) Quantitative reverse transcription PCR verifications of the up‐regulated genes in *B. cinerea*. *BcCGF1*, *B. cinerea* conidial germination factor 1. *BcBGL*, *B. cinerea* β‐glucosidase genes. (b) The up‐regulated genes encoding putative β‐glucosidases play a limited role in the virulence of *B. cinerea*. The indicated β‐glucosidase genes were separately deleted in *B. cinerea* and the resultant mutants, together with the wild‐type strain B05.10, were used for pathogenicity assays. (c) Loss of the up‐regulated gene *BCIN_03g01540* encoding GMC oxidoreductase significantly reduced the fungal virulence. (d) Quantification of the lesion sizes caused by the indicated strains as shown in (c). Data represent means ± *SD* from at least four independent experiments. ***Significance at *p* < .001

Each DEG with unknown function may constitute a potential entry point for investigating the novel mechanism by which the pathogen secures a successful infection. To investigate gene function at early stage of infection, we representatively selected several up‐regulated genes (with known and unknown function) and performed quantitative reverse transcription PCR (RT‐qPCR) to confirm their expression levels (Figure 2a). We then generated gene knockout (KO) mutants as previously described (Feng *et al.*, 2017) and conducted pathogenicity assays. Consistent with previous findings (Schumacher *et al.*, 2015; Ren *et al.*, 2017), the mutants lacking *BcATG1*, *BcLAE1*, or *BcVEL1* lost their virulence (Figure S5). However, loss of individual β‐glucosidase genes *BcBGL1* through *BcBGL6* (*BCIN_09g02640*, *BCIN_09g05460*, *BCIN_10g05590*, *BCIN_03g08710*, *BCIN_14g00650*, and *BCIN_10g02650*) did not affect the virulence of the pathogen (Figure 2b), implying that those CWD‐associated factors may be functionally redundant in virulence and/or act on modulating fungal cell walls during growth and development of the grey mould fungus. Disruption of *BCIN_03g01540*, a gene encoding a glucose‐methanol‐choline (GMC) oxidoreductase, significantly reduced the fungal virulence (Figure 2c,d), suggesting that the factor is required for a successful infection.

### 2.5 *BcCGF1 *is a novel virulence‐associated gene required for virulence of *B. cinerea*

Among the fungal DEGs, we were particularly intrigued with these novel DEGs; *BCIN_16g01820*, encoding a hypothetical protein (Table 2), represented one such DEG because it had not been previously implicated as a factor that mediates fungal pathogenesis. We thus focused on the functional analysis of this DEG. Bioinformatics analysis indicated that the hypothetical protein contained 255 amino acid residues and shared 66% identity with the spore germination protein (PQE13889.1) of *Rutstroemia* sp. NJR‐2017a BBW (Figure S3); however, the protein has not been previously characterized. Our functional analysis demonstrated that *BCIN_16g01820* was involved in *B. cinerea* conidial germination (see below), therefore *BCIN_16g01820* was designated as *B. cinerea* conidial germination‐associated factor 1 (*BcCGF1*). BcCgf1 is conserved among some known fungi and putatively conserved domains have not been detected in the protein (Figure S3).

**FIGURE 3:**
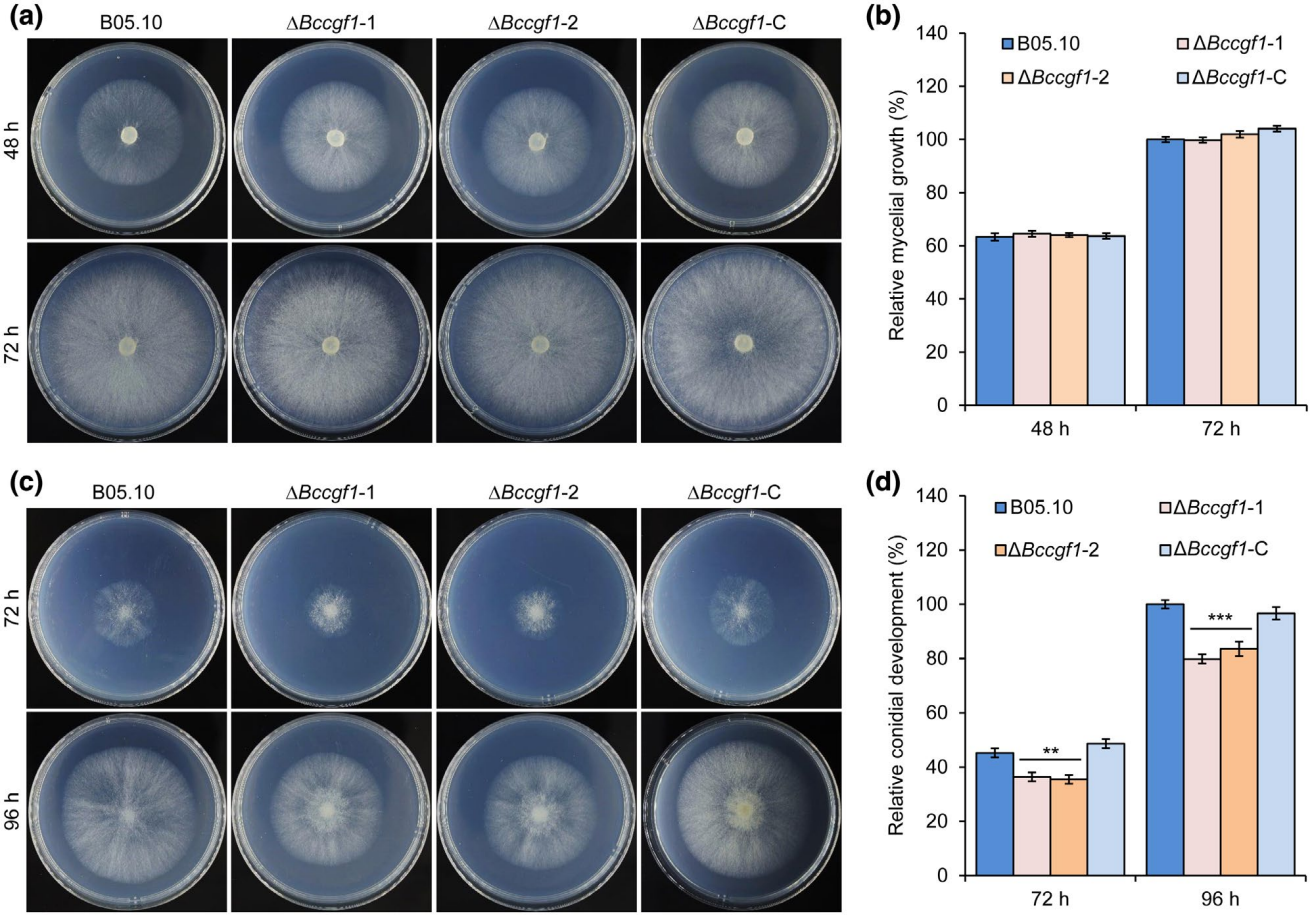
*BcCGF1* is required for *Botrytis cinerea* conidial germination but not hyphal radial growth. Mycelium‐plug radial growth of the indicated wild type (B05.10), Δ*Bccgf1*, and complemented (Δ*Bccgf1‐*C) strains of *B. cinerea* on complete medium (CM) plates at 20 °C with a time course of 72 hr (a) and quantification of the mycelial growth (in diameter) at 72 hr post‐inoculation (hpi) (b). Conidial growth of the indicated strains on CM plates at 20 °C with a time course of 96 hr (c) and quantification of the growth at 96 hpi (d). Data represent means ± *SD* from at least four independent experiments. **, ***Significance at *p* < .01 and *p* < .001, respectively

**FIGURE 4:**
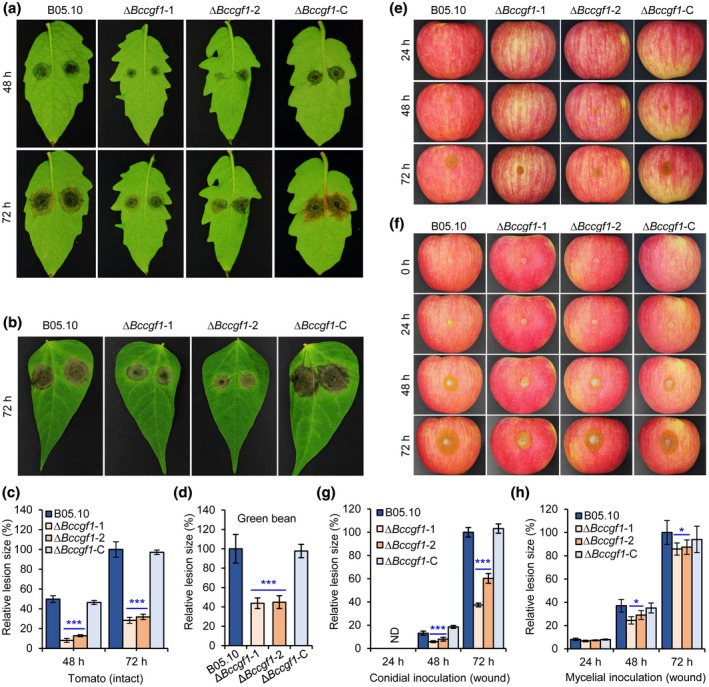
*BcCGF1* is required for virulence of *Botrytis cinerea*. Host leaves and fruits were inoculated, incubated at room temperature in the dark, and at the indicated times after inoculation the inoculated materials were photographically documented and quantitatively analysed. Intact leaves were inoculated with the indicated strains via the conidial inoculation approach (10^5^ conidia/ml, 8 μl) and the diseased tomato (a) or green bean (b) leaves at the indicated times after inoculation were photographically documented. Quantification of the relative lesion sizes on inoculated tomato (c) and green bean (d) leaves. Diseased apple fruits induced by conidia (10^5^ conidia/ml, 5 μl) (e) or mycelial plugs (f) of the indicated strains at the indicated times after inoculation and quantification of the relative lesion sizes induced by conidia (g) or mycelial plugs (h) of the strains as shown in (e) and (f). Data represent means ± *SD* from at least four independent experiments. *, ***Significance at *p* < .05 and *p* < .001, respectively

To analyse the roles of *BcCGF1* in the fungal growth and pathogenesis, we generated *B. cinerea CGF1* knockout (KO) mutant Δ*Bc*cgf1 and its complemented strain Δ*Bc*cgf1*‐*C using the illustrated strategies and performed functional analyses after confirmation of the absence of *BcCGF1* in the mutants (Figure S4). Our data indicated that loss of *BcCGF1* in *B. cinerea* did not affect mycelial growth (Figure 3a,b), but reduced conidial development (Figure 3c,d) on complete medium (CM) and disruption of *BcCGF1* impaired virulence of the Δ*Bc*cgf1 mutants on both intact (Figure 4a–d) and wounded (Figure 4e–h) hosts. A similar result was observed when mycelial plugs of the tested strains were used to inoculate green bean leaves (Figure S6). Complementation of the mutants with the wild‐type (WT) *CGF1* allele completely rescued the defects of the mutants (Figures 3 and 4). The findings demonstrate that *BcCGF1* is a novel virulence‐associated factor required for conidial development, virulence, and in planta development of the pathogen.

### 2.6 *BcCGF1 *is required for *B. cinerea* IFS development and host penetration

**FIGURE 5:**
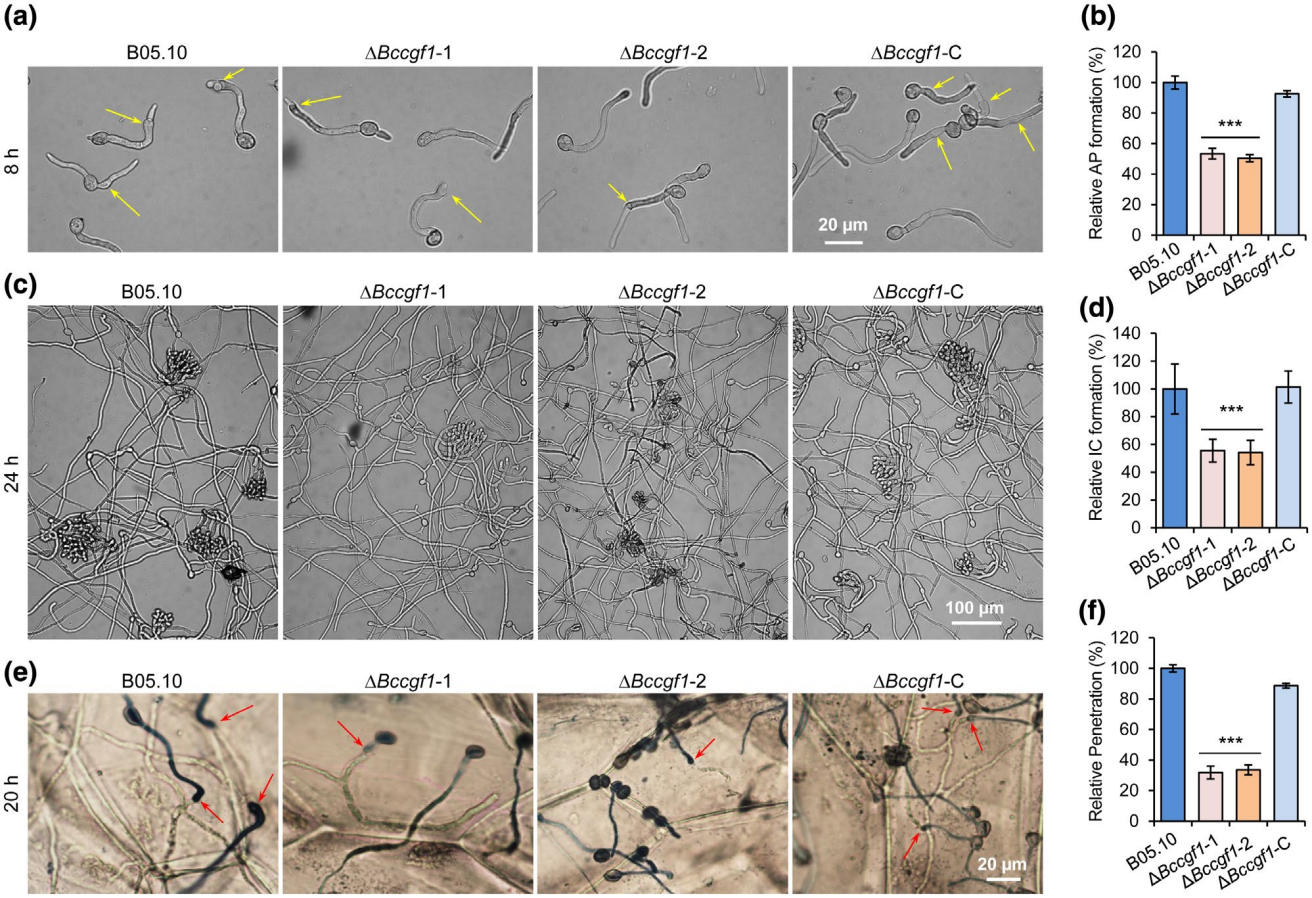
*BcCGF1* mediates infection structure formation and host penetration. Disruption of *BcCGF1* reduces appressorium (arrows) (a) and infection cushion (c) formation of the indicated strains on an inductive surface at 8 or 24 hr post‐inoculation (hpi) at 20 °C. Quantification of appressorium (b) and infection cushion (d) formation by the indicated strains at 8 and 24 hpi, respectively. (e) Loss of *BcCGF1* impairs host penetration and invasive growth of the mutant strains. (f) Quantification of host penetration by the indicated strains at 20 hpi. Data represent means ± *SD* from three independent experiments with triplicate samples/slides examined for each strain in each experiment. ***Significance at *p* < .001

To investigate the reduction in virulence of the Δ*Bccgf1* mutants, we evaluated the capability of the WT, Δ*Bccgf1*, and Δ*Bcgf1*‐C strains to form IFSs that play crucial roles in pathogen host penetration. We inoculated the tested strains on inductive surfaces (Feng *et al.*, 2017) and determined their abilities to form appressoria and infection cushions. Our data demonstrated that loss of *BcCGF1* reduced the pathogen formation of appressoria (Figure 5a,b) and infection cushions (Figure 5c,d). To further understand the attenuated pathogenicity of the Δ*Bccgf1* mutants, we performed onion epidermal cell infection assay to analyse host penetration of the strains and found that at 20 hr post‐inoculation/incubation (hpi), almost all the WT and Δ*Bc*c*gf1‐*C conidia penetrated the onion epidermis; host penetration by the Δ*Bccgf1* mutants was only 32% of that of the control (Figure 5e,f). These findings demonstrate that *BcCGF1* is required for the pathogen IFS formation and host penetration.

### 2.7 *BcCGF1 *mediates *B. cinerea* conidiation, conidial morphogenesis, and germination, but is dispensable for sclerotial formation and germination

**FIGURE 6:**
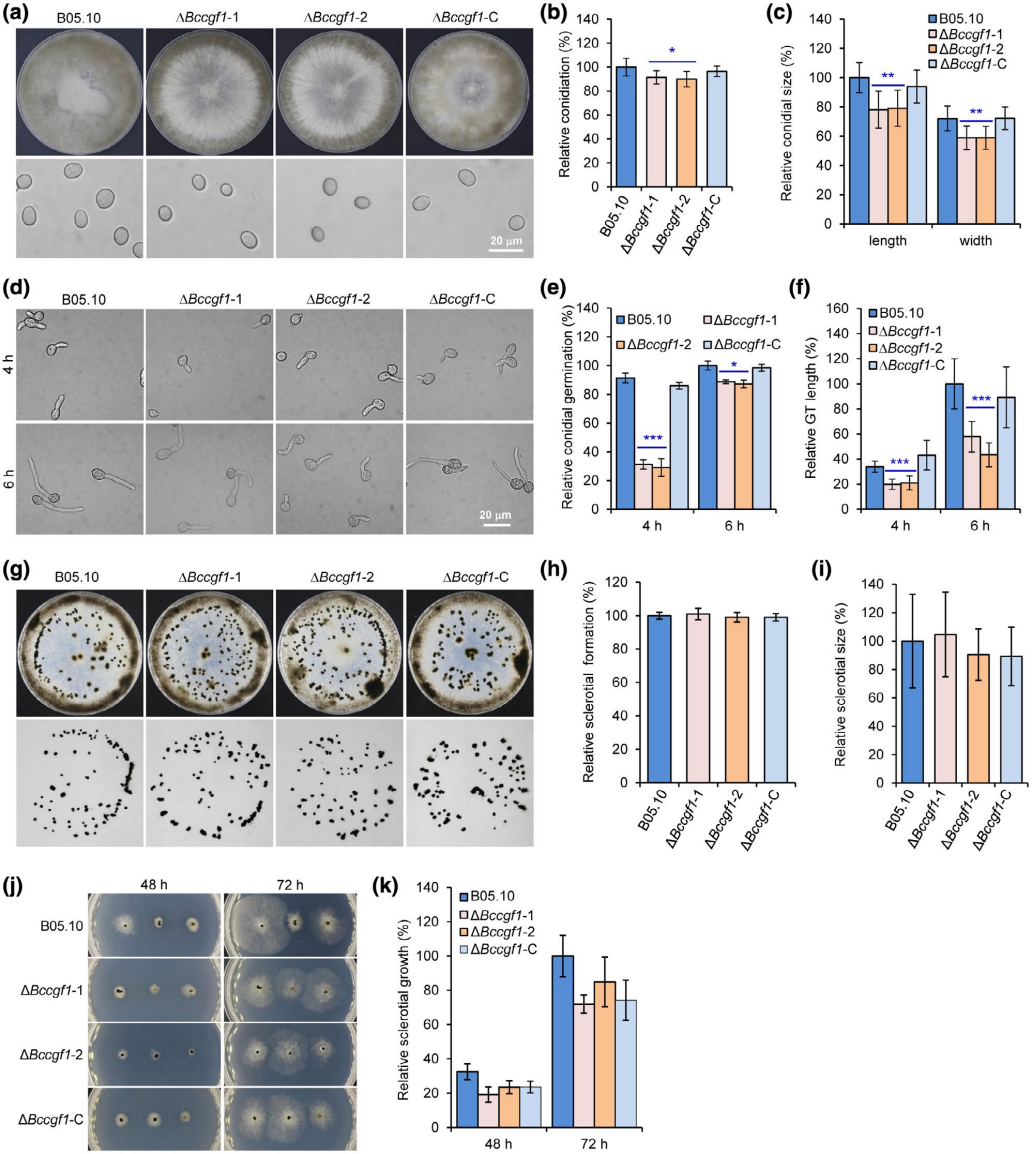
*BcCGF1* is required for proper *Botrytis cinerea* conidiation, conidial morphogenesis, and germination but is dispensable for *B. cinerea* sclerotium production and morphogenesis. (a) Loss of *BcCGF1* reduces *B. cinerea* conidiation (upper panel) and alters conidial morphology (lower panel). Quantification of conidiation (b) and conidial size (c) of the indicated strains. (d) Conidial germination of the indicated strains. Quantification of conidial germination (e) and germ tube (GT) length (f) at 4 and 6 hr post‐inoculation (hpi). Deletion of *BcCGF1* in *B. cinerea* did not affect sclerotial production. The wild type (B05.10), Δ*Bccgf1*, and Δ*Bccgf1*‐C strains were inoculated on complete medium (CM) plates at 20 °C in darkness after 14 days. Sclerotial production by each strain was photographically documented (g) and quantitatively determined (h). (i) Quantification of the sizes of sclerotia produced by the indicated strains at 14 days. (j) Sclerotial germination of the indicated strain on CM plates at the indicated hpi. (k) Relative sclerotial growth of the indicated strains (nine sclerotia/plate). Data represent means ± *SD* from at least three independent experiments in which triplicate plates were analysed for each strain in each experiment. *, **, *** significance at *p* < .05, *p* < .01, and *p* < .001, respectively

To determine the roles of *BcCGF1* in fungal development, we inoculated the WT, ∆*Bccgf1*, and ∆*Bccgf1*‐C strains on glass slides or CM plates and performed fungal development assays. Disruption of *BcCGF1* reduced conidiation of the ∆*Bccgf1* mutants (Figure 6a,b). The mutant conidia displayed an abnormal morphology (Figure 6a, lower panel). Quantitative analysis demonstrated that conidia produced by the mutants were smaller in both length and width than those produced by the WT and complemented strains (Figure 6c). The subsequent conidial germination assay indicated that disruption of *BcCGF1* reduced the conidial germination rate and germ tube development (Figure 6d–f). Complementation of the mutants with the WT *BcCGF1* rescued the defects (Figure 6). However, loss of *BcCGF1* did not impair the production, morphology, or germination of the mutant sclerotia (Figure 6g–k). These findings indicate that *BcCGF1* is required for conidiation, conidial morphogenesis and germination, and germ tube development, but dispensable for sclerotium formation and germination, of the pathogen.

### 2.8 *BcCGF1* mediates *B. cinerea* osmotic and oxidative stress adaptation as well as cell wall integrity

**FIGURE 7:**
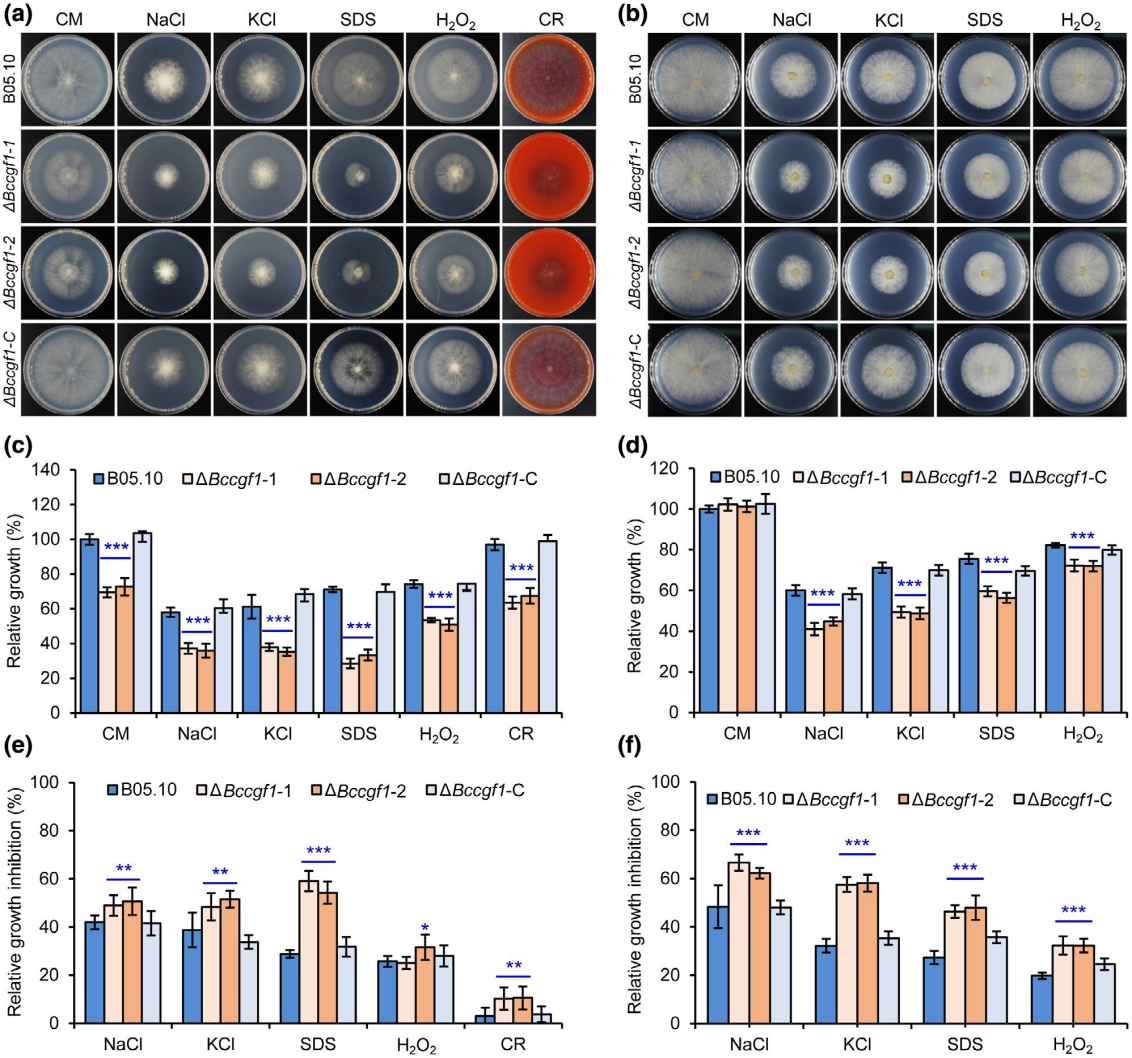
*BcCGF1* mediates osmotic and oxidative stress adaptation as well as cell wall integrity of *Botrytis cinerea*. Conidial (5 × 10^5^ conidia/ml, 5 μl) (a) and mycelial radial growth (mycelial plugs 5 mm in diameter) (b) of the wild type (B05.10), Δ*Bccgf1*, and Δ*Bccgf1*‐C strains of *B. cinerea* on complete medium (CM) supplemented with the osmotic stress agents NaCl (1 M) and KCl (1 M), the oxidative stress agent H_2_O_2_ (6 mM), the cell wall disturbing agents sodium dodecyl sulphate (SDS, 0.01%) or Congo Red (CR, 300 μg/ml). Quantification of the relative mycelial growth of the indicated strains growing from inoculated conidia (c) or mycelial plugs (d) on CM supplemented with the indicated stress‐mimetic agents as presented in (a) and (b). The relative mycelial growth inhibition of the indicated strains growing from inoculated conidia (e) or mycelial plugs (f) on CM supplemented with the indicated stress‐mimetic agents as presented in (a) and (b). Representative images are from one experiment (4 days post‐inoculation). Data represent means ± *SD* from three independent experiments in which triplicate plates were examined for each strain in each experiment. *, **, *** significance at *p* < .05, *p* < .01, and *p* < .001, respectively

To test whether *BcCGF1* plays a role in *B. cinerea* adaptation to infection‐related stresses, we inoculated the WT, Δ*Bccgf1*, and complemented strains on CM supplemented with the assorted stress‐mimicking agents, including osmotic stress agents NaCl and KCl, the oxidative stress agent H_2_O_2_, and the cell wall‐disturbing agents sodium dodecyl sulphate (SDS) and Congo Red (CR) (Feng *et al.*, 2017), and compared the radial growth rates of these strains. The mutant strains displayed reductions in conidial germination and subsequent hyphal growth (Figure 7a,c) and reductions in radial growth of mycelia on CM containing the assorted stress agents (Figure 7b,d). Further analysis indicated that the relative mycelial radial growth inhibition (Cao *et al.*, 2018; Hou *et al*., 2020) of the Δ*Bccgf1* strains significantly increased when cultured on CM containing the stress‐mimicking agents compared to that of the control and complemented strains (Figure 7e,f). These data suggest that *BcCGF1* is required for pathogen osmotic and oxidative stress adaptation as well as cell wall integrity*.*

### 2.9 Loss of *BcCGF1* impairs endogenous ROS production and cAMP rescues the infection‐related development in the *BcCGF1* deletion strains

**FIGURE 8:**
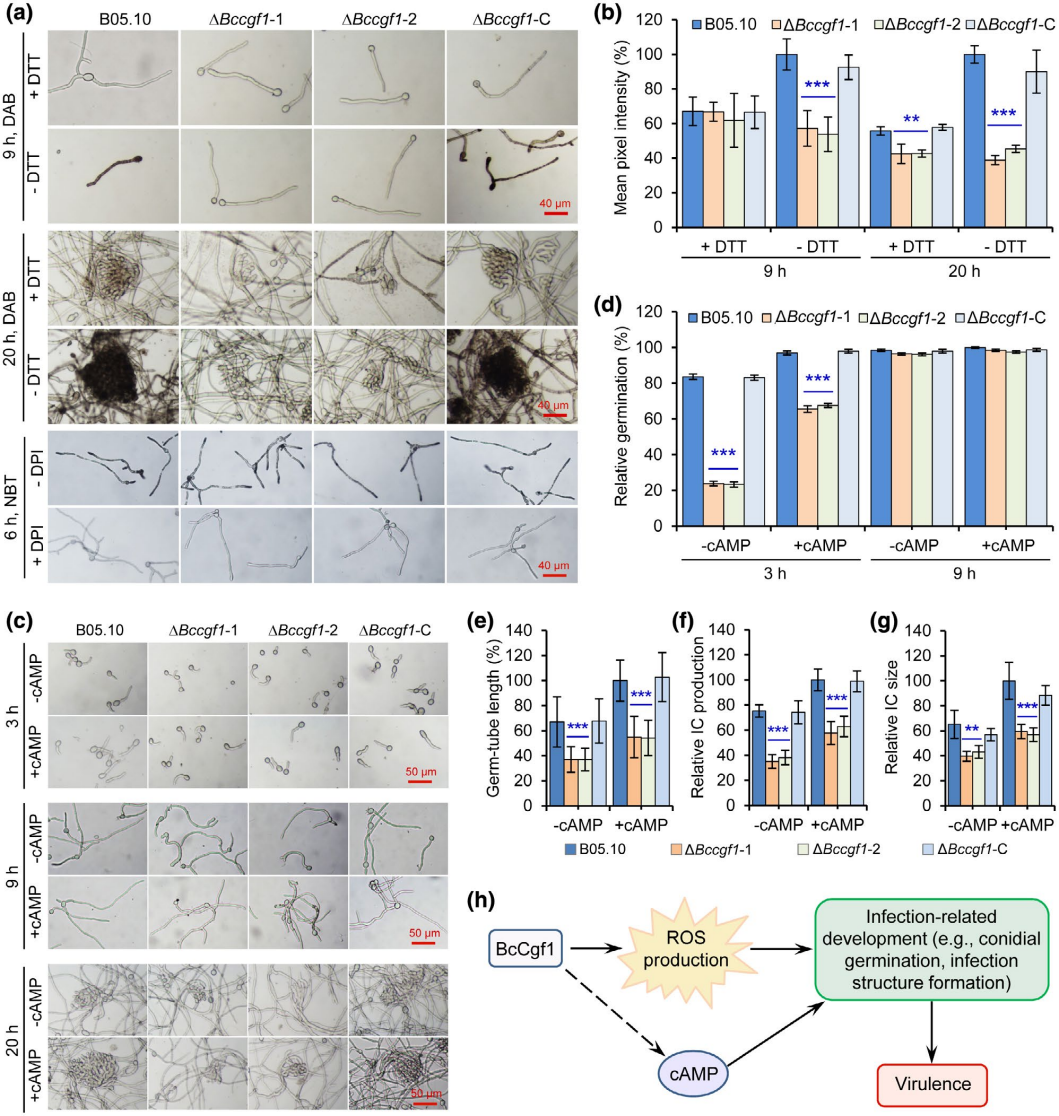
*BcCGF1* regulates endogenous reactive oxygen species (ROS) production in *Botrytis cinerea* and cAMP rescues the infection‐related development in the *BcCGF1* deletion strains. Conidia of the wild type (B05.10), ∆*Bccgf1*, and ∆*Bccgf1*‐C strains were cultivated on complete medium (CM) or CM supplemented with diphenylene iodonium (DPI) or dithiothreitol (DTT) at 20 °C for the indicated lengths of time, and then the germ tubes or hyphae/mycelia of the tested strains were stained with 3,3′‐diaminobenzidine (DAB) or nitroblue tetrazolium (NBT) solution as previously described (Cao *et al.*, 2018; Hou *et al*., 2020). (a) Disruption of *BcCGF1* reduced ROS production in the ∆*Bccgf1* mutants during infection‐related development detected by DAB or NBT staining. (b) Quantification of relative ROS production by the mean pixel intensity in the tested strains shown in (a). (c) Exogenous cAMP rescued the defect of infection‐related development of the mutants. Quantification of conidial germination (d), germ tube length (e), infection cushion (IC) production (f), and infection cushion size (g) of the tested strains supplemented with or without exogenous cAMP. (h) A model describing BcCgf1 regulation of *B. cinerea* pathogenesis via mediating infection‐related development and virulence controlled by BcCgf1‐mediated ROS production. Data represent means ± *SD* from three independent experiments. **, *** significance at *p* < .01 and *p* < .001, respectively

Endogenous ROS and the cAMP signalling pathway play crucial roles in *B. cinerea* infection‐related development, including conidial germination and IFS formation (Marschall and Tudzynski, 2016b; Feng *et al.*, 2017; Hou *et al*., 2020). Our data indicated that disruption of *BcCGF1* impairs the pathogen conidial germination, IFS formation, host penetration, and virulence (Figures 3, 4, 5, 6). We thus wondered whether loss of *BcCGF1* reduces endogenous ROS production and the cAMP signalling regulates these processes in *BcCGF1*‐deficient *B. cinerea*. To test these possibilities, we examined ROS production in the presence or absence of diphenylene iodonium (DPI), a NAD(P)H oxidase inhibitor, or antioxidant dithiothreitol (DTT), which prevents oxidation of 3,3′‐diaminobenzidine (DAB), in the tested strains using the DAB and nitroblue tetrazolium (NBT) staining approaches (Cao *et al.*, 2018; Hou *et al*., 2020). DAB is converted to dark‐brown polymers in the presence of H_2_O_2_ and NBT forms a dark‐blue water‐insoluble formazan precipitate on reduction by superoxide radicals. We also determined the influence of cAMP signalling on conidial germination and/or IFS formation of the tested strains as previously described (Feng *et al.*, 2017; Cao *et al.*, 2018; Hou *et al*., 2020). Our data demonstrate that the ∆*Bccgf1* mutants are difficult to be stained in the germ‐tubes and growing mycelia during conidial germination, appressorium, and infection structure formation (Figures 8a and S7); quantification analysis indicated that the relative ROS production by the mutants was significantly reduced (Figure 8b). These findings demonstrate that loss of *BcCGF1* in *B. cinerea* impairs endogenous ROS production.

The analysis of cAMP influence on the fungal infected‐related development indicated that exogenous cAMP (50 µM) significantly facilitates conidial germination, appressorium, and IFS formation of the mutants compared to that of the strains in the absence of cAMP (Figure 8c–g). Consistent with the previous reports (Feng *et al.*, 2017; Hou *et al*., 2020), exogenous cAMP in the WT and Δ*Bccgf1*‐C strains resulted in a promotion of conidial germination, and an earlier emergence and facilitation of IFS development (Figure 8c–g). These data suggest that cAMP signalling regulates the pathogen infection‐related development downstream of BcCgf1.

## 3 DISCUSSION

In this study, we performed simultaneous transcriptome analyses of tomato and *B*. *cinerea* during their early stage of interaction and identified 720 and 621 DEGs in tomato and *B*. *cinerea*, respectively. We used *B. cinerea* WT strain cultured in liquid medium, growing from spore suspension as a control. This culture condition can mimic the fungal infection environment inside host cells/tissues that may be hypoxic, and thus can minimize the interference of fungal genes that respond only to low‐oxygen conditions (Kawahara *et al.*, 2012). After DEG enrichment analysis, we identified the tomato and *B. cinerea* genes and pathways that may play roles in their early stage of interaction and functionally investigated the gene functions of some fungal DEGs, including *BcCGF1*.

Similar to the previous findings (Blanco‐Ulate *et al.*, 2013; Smith *et al.*, 2014; Vega *et al.*, 2015; Rezzonico *et al.*, 2017), we identified a large number of up‐regulated tomato genes that may increase plant defence against *B. cinerea.* These genes are mainly associated with ROS and phytohormone production, stress responses, and PR proteins, and so on (Tables 1 and S3). The balance between two types of programmed cell death, autophagy and apoptosis, controls host cell life, and an induction of autophagic cell death is conducive to triggering host cell resistance to *Botrytis* infection (Veloso and van Kan, 2018). The up‐regulation of *SlATG8* suggests that host autophagy machinery functions in tomato response to *B. cinerea* infection and cell survival.

Some new plant genes that may be involved in tomato plant resistance to *B. cinerea* attack were detected in our transcriptome analyses. Nine tomato ABC transporter‐related genes encoding protein members of diverse ABC transporter families were up‐regulated when infected by *B. cinerea* (Tables S3 and S11)*.* These ABC transporters may interact with other ABC transporter‐related proteins to fulfil complicated biological processes involved in tomato defence against *B. cinerea* (Figure 1a and Table S11). The plant JA signalling pathway is activated mainly against necrotrophic pathogens (El Oirdi *et al.*, 2011). The up‐regulation of *Solyc12g009220.1* (JAZ2) (Table S3), encoding TIFY 10A in the JA pathway, may promote JA biosynthesis in the infected tomato. The calmodulin‐binding transcription activators (CAMTAs) are crucial in manipulating both biotic and abiotic stresses in plants by their efficiency to transduce calcium signals that mediate plant cell wall reinforcement, stomatal closure, and activation of defence‐related genes (Galon *et al.*, 2010). Several tomato genes, for example *Solyc01g105230.2* (*LOC101055571*), *Solyc10g074570.1* (*LOC101252935*), *Solyc02g083850.2* (*LOC101260391*), and *Solyc06g073830.1* (*LOC101265138*) (Table S3), encoding CAMTAs or calcium‐related factors, were up‐regulated. The up‐regulation of these new tomato genes suggests that they may be required for tomato defence against pathogen attack or maintenance of survival of the infected cells.

Autophagy facilitates programmed cell developmental changes that occur during cellular remodelling, and thus serves as an adaptive mechanism for cellular nutrient starvation. Therefore, it is not surprising that some ATGs, including *BcATG1*, *BcATG2*, and *BcATG13*, were up‐regulated in the pathogen during initial infection. At early infection stage, *B. cinerea* may be in a condition of nutrient limitation and autophagy allows the pathogen to cope with nutrient starvation in the infection niches. The interactions of the autophagy proteins encoded by these ATGs with other autophagy‐related proteins in our PPI network analysis (Figure 1b, Table S12) suggest that the pathogen autophagy machinery is activated and functions during initial infection. The involvement of *B. cinerea* autophagy in securing a successful infection is likely via mediating its nutrition acquisition and development. These data also support the recent findings about the roles of autophagy in *B. cinerea* pathogenesis (Ren *et al.*, 2017, 2018a, 2018b; Liu *et al.*, 2019a).

Glucose metabolism is one of the basic characteristics of *B*. *cinerea* host infection. Glucose mediates pathogen development and pathogenesis via initiating conidial germination, mediating infection structure development, and host penetration (Liu *et al.*, 2018). To invade plant cells and/or exploit the polysaccharides of plant cell walls, the pathogen secretes diverse enzymes to disassemble plant cell walls during infection. The expression of fungal glycoside hydrolases contributes to the degradation of host cell wall polysaccharides, and the soluble oligosaccharides produced by the digestion of glycoside hydrolases are transported to the inside of the fungal cell and metabolized (Blanco‐Ulate *et al.*, 2014). Consistent with previous findings (Blanco‐Ulate *et al.*, 2014), our data indicate that many transcripts encoding enzymes in glycosyl hydrolase family (GHF), including GHF 11 (*BCIN_12g00090*), GHF 28 (*BCIN_10g06130*), GHF 45 (*BCIN_12g06630*), and GHF 76 (*BCIN_08g06110*), polysaccharide lyase family 6 (*BCIN_10g06130*), and pectate lyases (*BCIN_03g05820* and *BCIN_10g05620*) were highly up‐regulated in *B*. *cinerea* during initial infection (Table S5), suggesting that these enzymes may function in the pathogen CWD and break the plant physical barrier.

Unsurprisingly, a large number of genes encoding glucosidases were up‐regulated in *B. cinerea* during infection (Table S5). We generated the KO mutants of the six β‐glucosidase genes (*BcBGL1* to *BcBGL6*) with up‐regulation levels ranging from 3‐ to 26‐fold (Table S5) and performed pathogenicity assays for these mutants; however, disruption of these genes did not impair virulence of the mutants (Figure 2b), suggesting that these individual β‐glucosidases may be dispensable for virulence of the pathogen. This may result from the functional redundancy of these enzymes in *B. cinerea*, thus loss of one or two of them did not impair virulence of the pathogen.

Our functional analysis suggested that fungal GMC oxidoreductase BCIN_03g01540 is involved in *B. cinerea* host infection. The GMC superfamily is a large and functionally diverse family of oxidoreductases that share a common structural fold. Fungal GMC enzymes of this superfamily that exhibit lignocellulose degradation include aryl‐alcohol oxidoreductase, alcohol oxidase, cellobiose dehydrogenase, glucose oxidase, glucose dehydrogenase, pyranose dehydrogenase, and pyranose oxidase (Sutzl *et al.*, 2019). In polyporales, the members of the GMC oxidoreductase superfamily also play a central role in degradation of plant polymers because they generate extracellular H_2_O_2_, acting as the ultimate oxidizer in both white‐rot and brown‐rot decay (Ferreira *et al.*, 2015). The roles of GMC oxidoreductases in *B. cinerea* host infection remain largely unknown; further investigation should be focused on the mechanisms of GMC oxidoreductases regulating the fungal pathogenesis.

The up‐regulation of many new fungal genes in the early stage of *B. cinerea–*tomato interaction implies that they may play roles in facilitating the pathogen host infection. However, more research is needed to clarify the functions of these newly identified genes in the pathogenesis of *B. cinerea*. In this work, we functionally validated the roles of novel virulence‐associated gene *BcCGF1* in *B. cinerea*, identified by global mixed gene‐expression profiling. Bioinformatics analysis suggests that the deduced BcCgf1 may be a secreted protein containing a signal peptide, a transmembrane helix (TMhelix), and a noncytoplasmic domain (Figure S3a). Cgf1 proteins are evolutionarily conserved among pathogenic and nonpathogenic fungi (Figure S3b). However, the function of this protein has not been previously characterized. Disruption of *BcCGF1* impairs conidiation, alters conidial morphogenesis, dramatically delays conidial germination, and reduces infection structure formation, host penetration, and invasive hyphal growth of the mutant strains, which raises an intriguing question about how BcCgf1 influences these processes. Further mechanism analysis indicates that the pathogen infection‐related development is controlled by *BcCGF1*‐mediated ROS production, and cAMP regulation of the developmental events is probably downstream of BcCgf1 (Figure 8h). However, the signal that stimulates the up‐regulation of *BcCGF1* during infection remains unknown. Subcellular localization is an important functional characteristic of a protein, and subcellular localization of BcCgf1 needs to be determined, although bioinformatics analysis suggests that BcCgf1 may be a secreted protein (Figure S3a). The known functional domains of the deduced BcCgf1 are not detected (Figure S3a) and the functional characteristics of BcCgf1 also need to be further investigated. Further work is needed to address all the above‐mentioned issues to reveal the molecular mechanisms of BcCgf1 mediating the development and virulence of *B. cinerea*.

In summary, our global simultaneous transcriptome analyses of tomato and *B. cinerea* interaction at the early stage provides new insights into the mechanisms underlying tomato–*B. cinerea* interaction. We identified a novel fungal factor *BcCGF1* through the analyses and demonstrated that *BcCGF1* plays pleiotropic roles in *B. cinerea* conidial germination, asexual reproduction, infection structure formation, host‐penetration, stress adaptation, and virulence. *BcCGF1* enhances the fungal virulence via promoting infection‐related development controlled by *BcCGF1*‐mediated endogenous ROS production (Figure 8h).

## 4 EXPERIMENTAL PROCEDURES

### 4.1 Fungal and plant materials

*B. cinerea* WT B05.10 and its derived mutant and complemented strains were used in this study (Table S14). All strains were maintained on potato dextrose agar (PDA) or CM as previously described (Liu *et al.*, 2018). The plant materials used in this work included tomato, green bean, strawberry, and apple leaves or fruits.

### 4.2 Sample preparation for RNA‐Seq analysis

Tomato cultivar Moneymaker plants were grown in a growth chamber at 25 °C, 80% humidity, and a 14 hr:10 hr light/dark cycle. Seven‐week‐old plants (8–10 leaves/plant) were challenged with conidial suspension (10^6^ conidia/ml in potato dextrose broth [PDB]) of B05.10 by the spray‐inoculation method. Plants inoculated with ½ × PDB served as a mock‐inoculation control. The inoculated plants were kept in containers sealed with a sheet of transparent plastic film on each top to maintain high‐humidity infection environments. At 24 hpi, inoculated leaves were detached from each inoculated plant (four plants per group), the leaf samples were quickly rinsed (in ddH_2_O) and frozen in liquid nitrogen and stored at −80 °C for RNA extraction. In each experiment, each treatment contained three biological replicates (three groups of plants). Two independent experiments were completed for RNA‐Seq analysis. Conidia (10^6^ conidia/ml, 1 ml) of *B*. *cinerea* were inoculated into flasks containing 100 ml PDB and the flasks were incubated at room temperature with shaking (220 rpm). Homogenized mycelia were harvested at c.18 hpi and immediately snap‐frozen in liquid nitrogen and stored at –80 °C for RNA extraction.

### 4.3 RNA isolation and RNA‐Seq

Total RNAs were extracted using TRIzol reagent (Life Technologies, Shanghai, China). RNA quality was monitored on 1% agarose gels. RNA purity was determined by a NanoPhotometer spectrophotometer (Implen GmbH, München, Germany). RNA concentration was measured using a Qubit RNA Assay Kit in Qubit 2.0 Flurometer (Life Technologies). RNA integrity was assessed by the Bioanalyzer 2100 system (Agilent Technologies, Beijing, China). The extracted mRNA was used to construct the cDNA library and the library construction was sequenced on an Illumina Hiseq 2000 Platform (Novogene Bioinformatics Institute, Beijing, China). The preprocessed RNA‐Seq reads were mapped to the tomato or *B. cinerea* reference genome using the Bowtie, TopHat, and Cufflinks programs (Trapnell *et al.*, 2009; 2010).

### 4.4 Transcriptome analysis

The DEGs were identified from RNA‐Seq data with the cut‐off of corrected *p* value < .01. Analyses of the biological information of DEGs were performed as previously described (Hou *et al*., 2020). The database used in the analyses included EggNOG (evolutionary genealogy of genes: Non‐supervised Orthologous Groups, http://eggnogdb.embl.de/#/app/home), a database of orthologous groups of genes, Gene Ontology Consortium, an international standard classification system for gene function, including biological processes, cellular components, and molecular function (http://www.geneontology.org/), KEGG (http://www.genome.jp/kegg/), Swiss‐Prot (http://web.expasy.org/docs/swiss-prot_guideline.html), NR (ftp://ftp.ncbi.nlm.nih.gov/blast/db/), and Pfam (http://pfam.xfam.org/).

### 4.5 RT‐qPCR

Total RNA for RT‐qPCR assay was extracted using RNAiso plus kid (TaKaRa), and cDNA was prepared using PrimerScript RT Reagent Kit with gDNA Eraser (TaKaRa) according to the manufacturer's instructions. RT‐qPCRs were carried out as previously described (Feng *et al.*, 2017).

### 4.6 Bioinformatics analysis

The data of *BcCGF1* DNA and protein sequence were obtained from NCBI (http://www.ncbi.nlm.nih.gov) and Ensembl Fungi (http://fungi.ensembl.org/Botrytis_cinerea) (*B. cinerea* B05.10) (Amselem *et al.*, 2011; Van Kan *et al.*, 2017). The deduced protein domains and functional sites of BcCgf1 were analysed via InterProScan (http://www.ebi.ac.uk/interpro/scan.html)*.* Sequence alignments and phylogenetic trees were generated using GENEDOC software (http://www.softpedia.com/get/Science-CAD/GeneDoc.shtml) and MEGA 6 software (Tamura *et al.*, 2013), respectively. The PPI networks were analysed using the SRTING software (v. 11.0, https://string-db.org/) and generated by the Cytoscape software (https://cytoscape.org/).

### 4.7 Generation of gene deletion mutants and complemented strains

Generation of gene KO mutants (including Δ*Bccgf1*, Δ*Bclae1*, Δ*Bcvel1*, Δ*Bcatg1*, Δ*BCIN_03g01540*, and Δ*Bcbgl1* to Δ*Bcbgl6*) and the genetic complemented strain Δ*Bccgf1‐*C was performed as previously described (Feng *et al.*, 2017). Briefly, vector pXEH containing the *HPH* cassette was used for replacement of the targeted genes. The 5′ and 3′ homologous flanks of the targeted gene were amplified and cloned into pXEH upstream and downstream of *HPH*, respectively. The gene KO vector was transformed into *Agrobacterium tumefaciens* AGL‐1. The resultant deletion transformants were screened on PDA with 100 μg/ml hygromycin. Vector pSUL conferring resistance to chlorimuron‐ethyl was used for complementation of the ∆*Bccgf1*. A fragment containing *BcCGF1* (1219 bp upstream and 686 bp downstream of the coding region of *BcCGF1*) was amplified by PCR and cloned into pSUL vector to generate the complementation vector. The complementary vector was transformed into *A. tumefaciens* AGL‐1 and the resultant transformants were screened on defined complex medium containing 100 μg/ml chlorimuron‐ethyl (Rolland *et al.*, 2003). Diagnostic PCR was performed to verify the integration events of the selected transformants. The gene KO and complemented strains were further confirmed by RT‐qPCR (Weiberg *et al.*, 2013). The primers used in the experiments are listed in Table S15.

### 4.8 Fungal growth and pathogenicity assays

For growth assays, conidia of *B. cinerea* WT, gene KO, and complemented strains were harvested with PDB, and conidial suspension was adjusted to approximately 10^5^ conidia/ml. Conidial suspension (10 μl) was dropped onto glass slides to observe conidial morphology and germination, or onto CM plates to observe mycelial growth. Stress adaptation assays and the influence of cAMP on the development of the tested strains were performed as previously described (Feng *et al.*, 2017; Hou *et al*., 2020). For pathogenicity assay, droplets of conidial suspension (5 × 10^5^ conidia/ml in ½ × PDB, 5 μl) of the tested strains were dropped on host leaves/surfaces. When mycelial plugs were used in these assays, mycelial plugs (5 mm in diameter) taken from a 3‐day‐old culture of the tested strains were used, unless otherwise indicated. The inoculated slides or plant materials were incubated in the dark in a moistened box at 21 °C. Conidial germination, appressorium, and infection cushion formation were observed and counted under a microscope. At least 100 conidia were counted per replicate in each experiment. At least three independent experiments (triplicate samples examined for each treatment in each experiment) were performed. The assays were performed as previously described (Feng *et al.*, 2017; Cao *et al.*, 2018; Liu *et al.*, 2018).

### 4.9 Cytological assay

Preparation of conidia and onion epidermal cells as well as sample lactophenol blue staining were performed as previously described (Liu *et al.*, 2018, 2019b; Hou *et al*., 2020). The infected samples were microscopically observed, photographically documented, and analysed at 20 hpi.

### 4.10 Detection and quantification of ROS production

Detection and quantification of ROS production during conidial germination and infection structure formation were performed as previously described (Feng *et al.*, 2017; Cao *et al.*, 2018; Hou *et al*., 2020). The software ImageJ (http://rsbweb.nih.gov/ij/) was used to quantify ROS production.

### 4.11 Statistical analysis

The quantitative data in this study were derived from at least three independent experiments with triplicate treatments examined unless otherwise indicated. The data of controls, including conidial germination, mycelial growth, and lesion size, in each independent experiment were normalized as 1 or 100%. The significance between the data were assessed using the Student's *t* test and *p* < .05 was considered as a significant difference.

## AUTHOR CONTRIBUTIONS

Q.M.Q. and M.Z.Z. conceived the experiments. M.Z.Z., C.H.S., Y.L., H.G.F., S.N.C., and J.H. performed the experiments. Q.M.Q., G.H.L., K.Z.S., H.W.C., C.C., and H.Z. analysed and interpreted the data. Q.M.Q. and G.H.L. provided reagents. Q.M.Q. supervised the work. Q.M.Q. and M.Z.Z. wrote the paper.

## Supporting information


**FIGURE S1** Differentially expressed genes in tomato and *Botrytis cinerea *at the early stage of their interaction

**FIGURE S2** The 20 most enriched KEGG pathways in tomato (a) and *Botrytis cinerea *(b) during their early stage of interaction

**FIGURE S3** Phylogenetic relationship of Cgf1 proteins from the indicated organisms

**FIGURE S4** Strategies of generation of *BcCGF1* deletion and complemented strains

**FIGURE S5** Functional validation of the up‐regulated* Botrytis cinerea *differentially expressed genes *BcATG1*, *BcLAE1*, and *BcVEL1*

**FIGURE S6** Loss of *BcCGF1* reduces the virulence of *Botrytis cinerea*

**FIGURE S7** *BcCGF1* mediates endogenous reactive oxygen species production in *Botrytis cinerea*

**TABLE S1** Mapping results of RNA‐Seq reads

**TABLE S2** The percentages of the clean reads mapped to tomato and *Botrytis cinerea* genomes

**TABLE S3** Up‐regulated tomato genes in response to *Botrytis cinerea* attack (24 hr post‐infection)

**TABLE S4** Down‐regulated tomato genes in response to *Botrytis cinerea* attack (24 hr post‐infection)

**TABLE S5** Up‐regulated *Botrytis cinerea* genes at the early stage of host infection (24 hr post‐infection)

**TABLE S6** Down‐regulated *Botrytis cinerea* genes at the early stage of infection (24 hr post‐infection)

**TABLE S7** GO enrichment of the differentially expressed genes in tomato infected by *Botrytis cinerea *at 24 hr post‐infection

**TABLE S8** GO enrichment of the differentially expressed genes in *Botrytis cinerea *at 24 hr post‐infection

**TABLE S9** KEGG enrichment of the differentially expressed genes in tomato attacked by *Botrytis cinerea *at 24 hr post‐infection

**TABLE S10** KEGG enrichment of the differentially expressed genes in *Botrytis cinerea *at 24 hr post‐infection

**TABLE S11** ABC transporters encoded by the up‐regulated tomato genes in response to *Botrytis cinerea* infection (early stage) and their interacting proteins

**TABLE S12** Proteins encoded by the up‐regulated *Botrytis cinerea* autophagy genes at the early stage of infection and their interacting proteins

**TABLE S13** Potential secreted *Botryis cinerea* proteins at the early stage of host infection

**TABLE S14** Fungal strains used in this study

**TABLE S15** Primers used in this study
