# Supplementary material for: Transcriptome analysis and functional validation reveal a novel gene, BcCGF1, that enhances fungal virulence by promoting infection‐related development and host penetration
Source: Mol Plant Pathol. 2020 Apr 16;21(6):834–53. doi: 10.1111/mpp.12934 (PMC7214349; doi:10.1111/mpp.12934)
Supplement: Supplementary file 1 — FIGURE S1 Differentially expressed genes in tomato and Botrytis cinerea at the early stage of their interaction [file MPP-21-834-s001.docx]

**
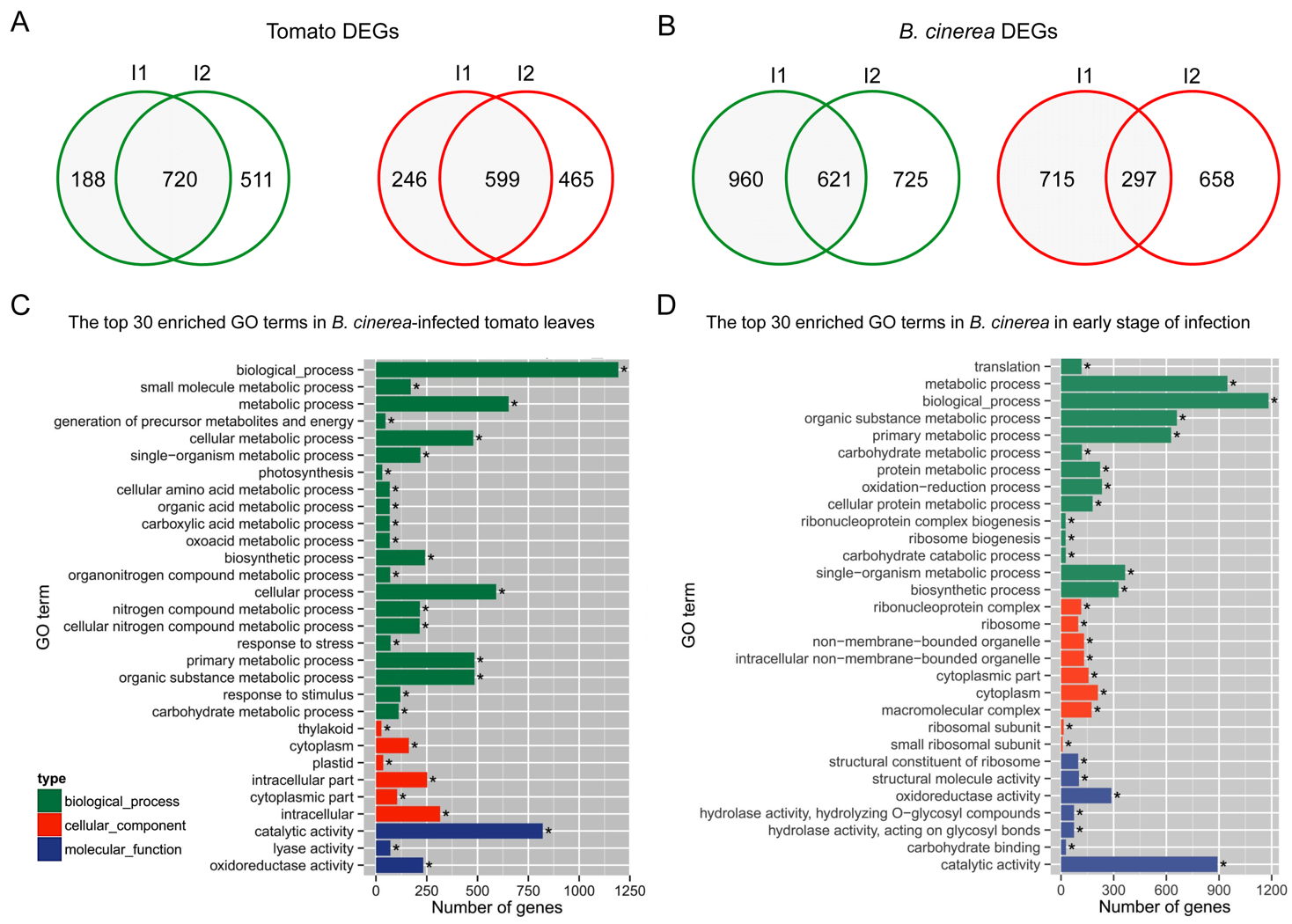
**

**Figure S1.** **Differentially expressed genes (DEGs) in tomato and** ***B. cinerea* at early stage of their interaction.** (A, B) Significantly upregulated and downregulated genes in tomato (A) and *B. cinerea* (B) during their early stage of interaction. (A) Tomato upregulated (left) and downregulated (right) genes during interaction with *B. cinerea*. (B) *B. cinerea* upregulated (left) and downregulated (right) genes when the fungus interaction with tomato leaves at early stage at 24 hpi. DEGs were filtered with a cut-off of |log2-fold change| ≥ 1 and FDR-corrected *p* ≤ 0.01. (C-D) The 30 most enriched GO terms in tomato (C) and *B. cinerea* (D) during their early stage of interaction. Bar charts in tomato (C) and *B. cinerea* (D) reflect the number of DEGs distributing into different GO terms. Data are from two independent experiments. I1 and I2: independent experiment 1 and 2, respectively.
