# Supplementary material for: Transcriptome analysis and functional validation reveal a novel gene, BcCGF1, that enhances fungal virulence by promoting infection‐related development and host penetration
Source: Mol Plant Pathol. 2020 Apr 16;21(6):834–53. doi: 10.1111/mpp.12934 (PMC7214349; doi:10.1111/mpp.12934)
Supplement: Supplementary file 2 — FIGURE S2 The 20 most enriched KEGG pathways in tomato (a) and Botrytis cinerea (b) during their early stage of interaction [file MPP-21-834-s002.docx]

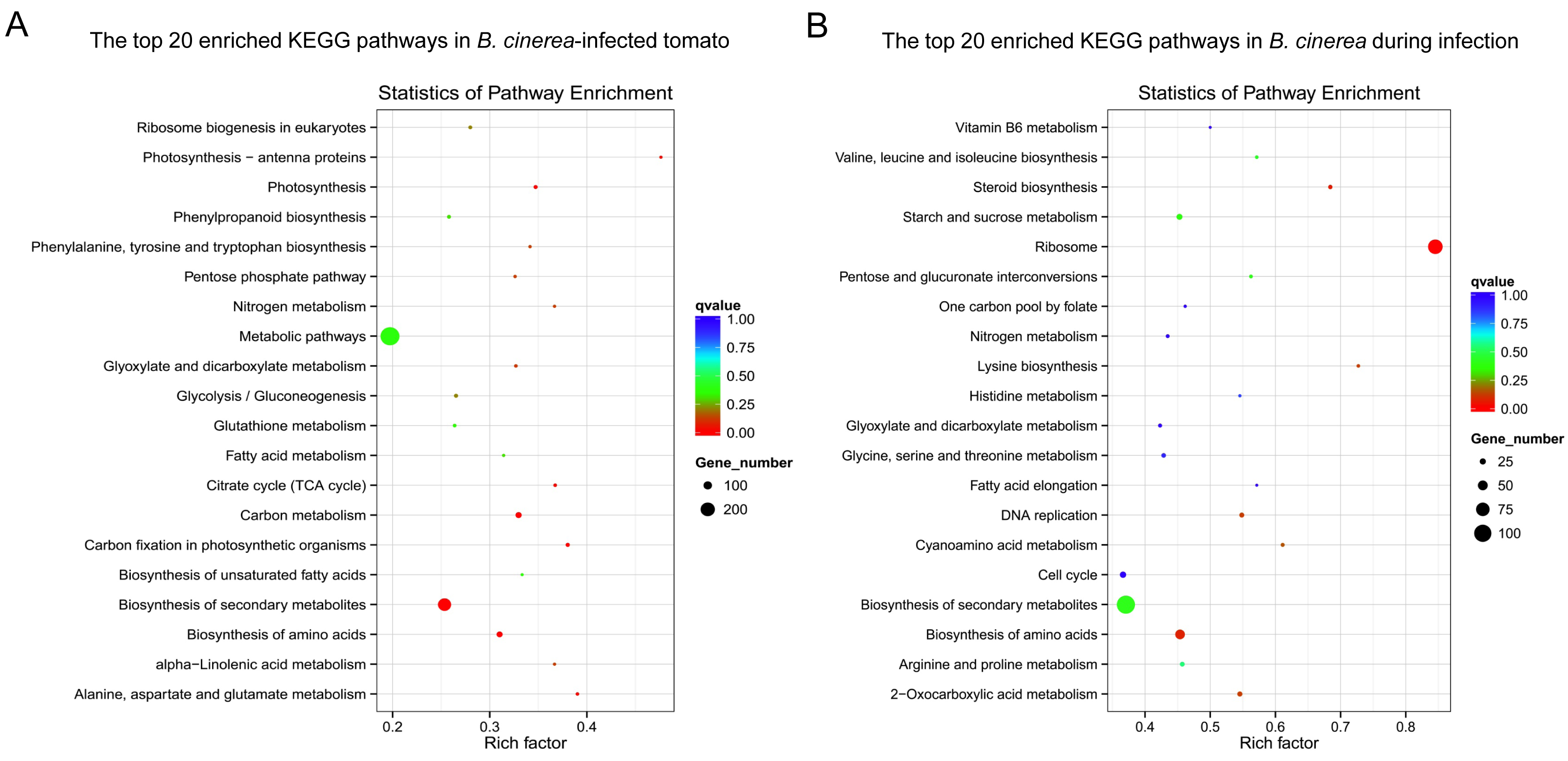


**Figure S2.** **The 20 most enriched KEGG pathways in tomato (A) and *B. cinerea* (B) during their early stage of interaction.** “Rich factor” represents the ratio of the DEGs number to the number of genes annotated in this pathway. The greater of the Rich factor, the greater the degree of enrichment tomato (A) or *B. cinerea* (B).
