# Supplementary material for: Transcriptome analysis and functional validation reveal a novel gene, BcCGF1, that enhances fungal virulence by promoting infection‐related development and host penetration
Source: Mol Plant Pathol. 2020 Apr 16;21(6):834–53. doi: 10.1111/mpp.12934 (PMC7214349; doi:10.1111/mpp.12934)
Supplement: Supplementary file 3 — FIGURE S3 Phylogenetic relationship of Cgf1 proteins from the indicated organisms [file MPP-21-834-s003.docx]

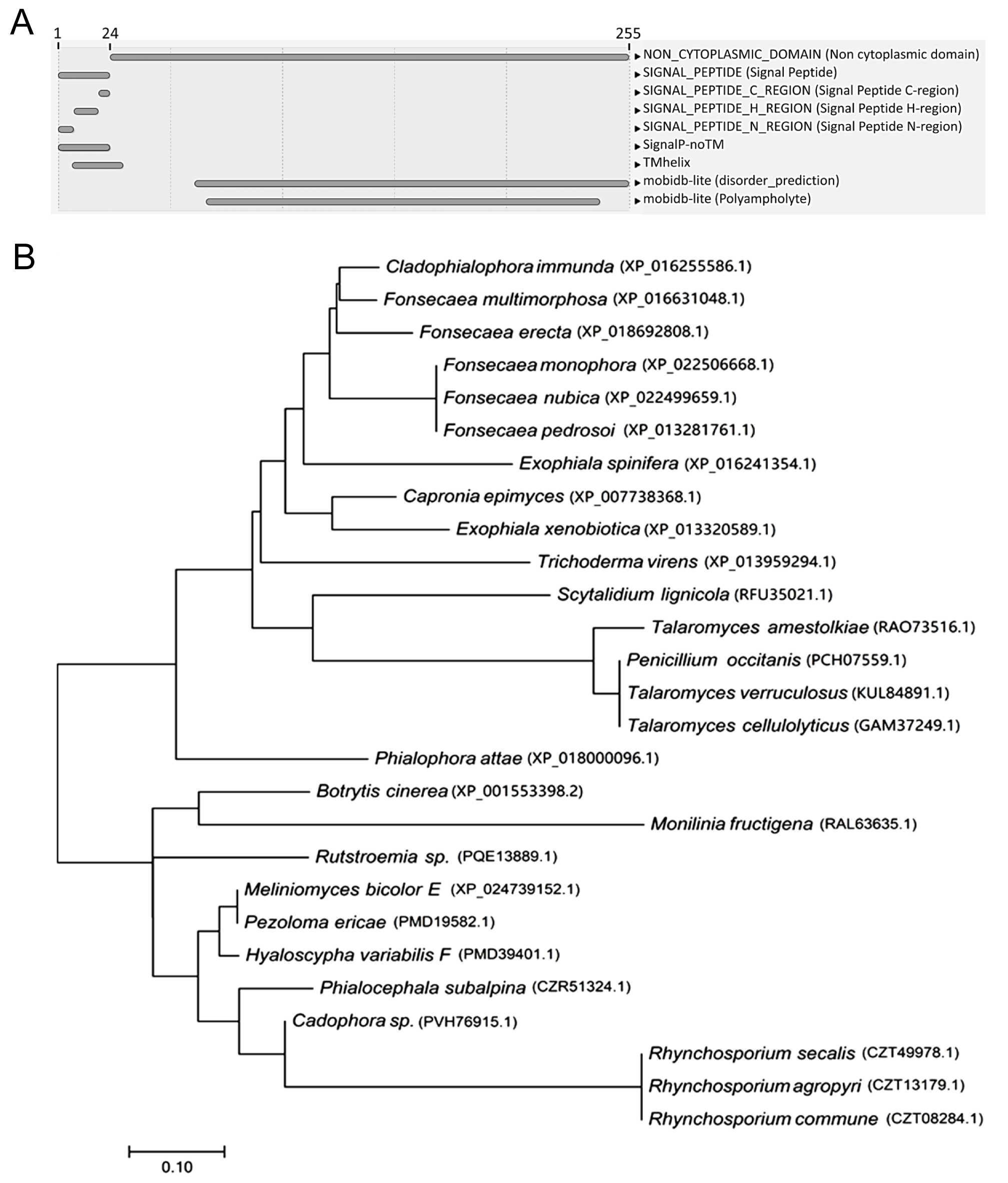


**Figure S3**. **Phylogenetic relationship of** **Cgf1 proteins from different fungal species.** (A) The deduced protein domains and functional sites of BcCgf1 based on InterProScan (http://www.ebi.ac.uk/interpro/scan.html) analyses. (B) Phylogenetic analysis of Cgf1 proteins from diverse fungi. The phylogenetic tree was generated using MEGA6 software with the neighbor-joining method and the maximum-likelihood distance composite model and 1,000 bootstrap replicates. The Cgf1 accessing numbers of the indicated fungal species are listed in the parentheses.
