# Supplementary material for: Transcriptome analysis and functional validation reveal a novel gene, BcCGF1, that enhances fungal virulence by promoting infection‐related development and host penetration
Source: Mol Plant Pathol. 2020 Apr 16;21(6):834–53. doi: 10.1111/mpp.12934 (PMC7214349; doi:10.1111/mpp.12934)
Supplement: Supplementary file 4 — FIGURE S4 Strategies of generation of BcCGF1 deletion and complemented strains [file MPP-21-834-s004.docx]

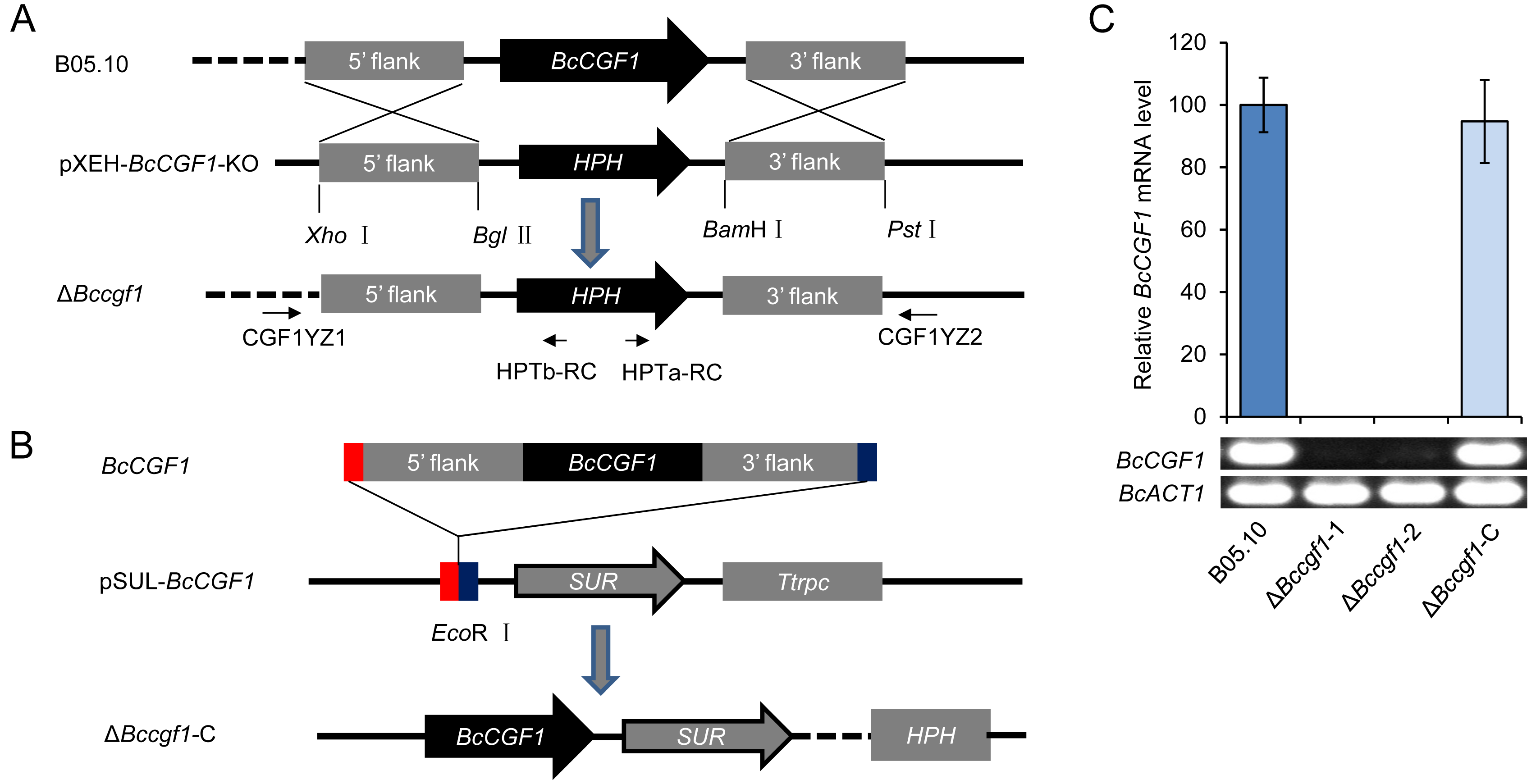


**Figure S4. Generation of the *BcCGF1* deletion mutants and the ∆*Bccgf1* complemented transformants.** (A) The gene-replacement strategies for generation of *BcCGF1* deletion mutant (∆*Bccgf1*)*.* (B) The strategy for generation of the *BcCGF1* deletion complemented (∆*Bccgf1*-C) strain. (C) Validation of the absence or presence of *BcCGF1* in the tested strains via real time quantitative RT-PCR (qRT-PCR).
