# Supplementary material for: Transcriptome analysis and functional validation reveal a novel gene, BcCGF1, that enhances fungal virulence by promoting infection‐related development and host penetration
Source: Mol Plant Pathol. 2020 Apr 16;21(6):834–53. doi: 10.1111/mpp.12934 (PMC7214349; doi:10.1111/mpp.12934)
Supplement: Supplementary file 5 — FIGURE S5 Functional validation of the up‐regulated Botrytis cinerea differentially expressed genes BcATG1, BcLAE1, and BcVEL1 [file MPP-21-834-s005.docx]

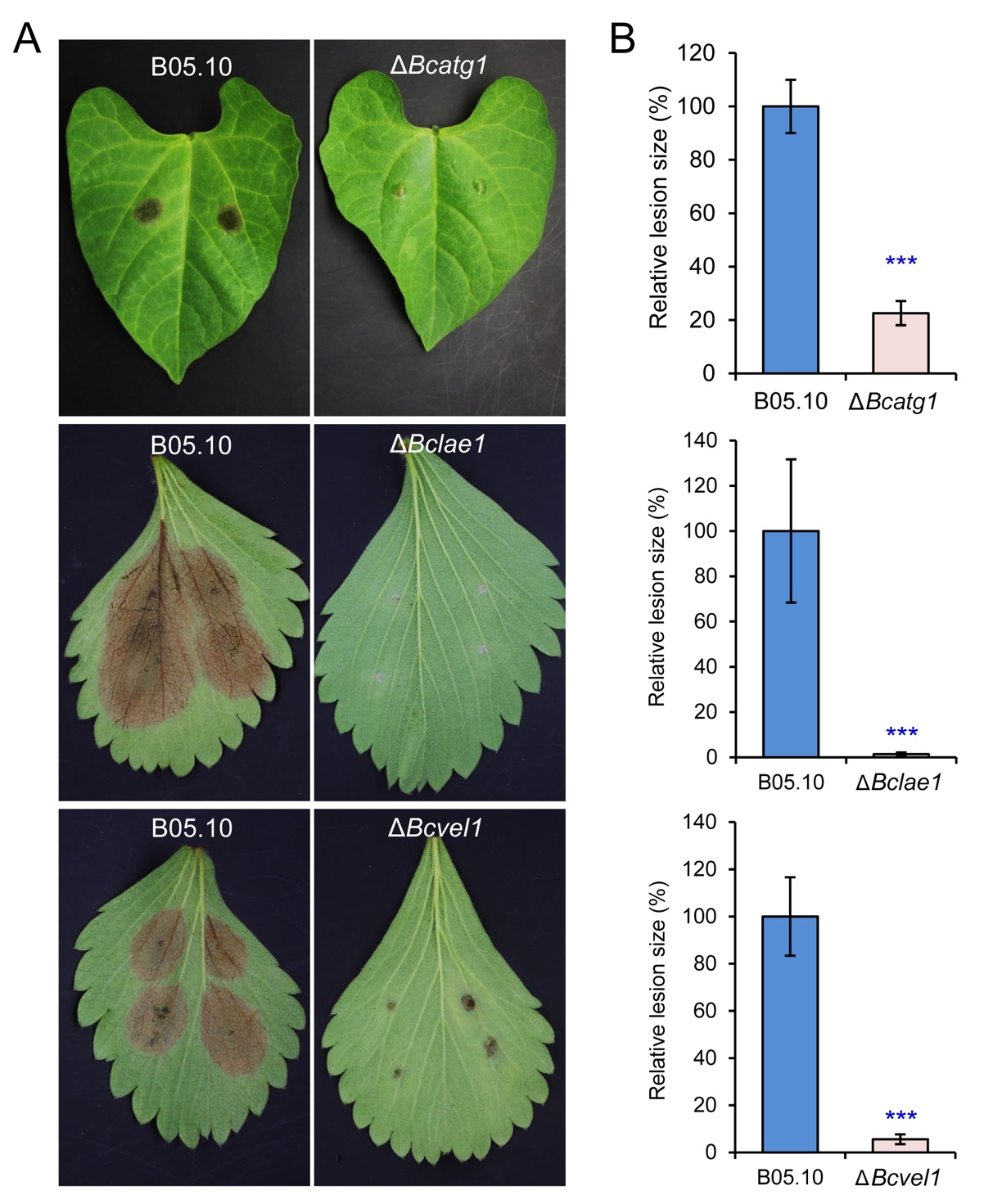


**Figure S5.** **Functional validation of the upregulated *B. cinerea* differentially expressed genes (DEGs) *BcATG1*, *BcLAE1* and *BcVEL1*.** (**A**) Conidial droplets (5 μl, 5×10^5^ conidia/ml) of the indicated B05.10 (wild-type strain), gene deletion mutant (∆*Bcatg1,* ∆*Bclae1* and ∆*Bcvel1*) strains were inoculated on the green bean or strawberry leaves and the diseased leaves were observed and photographically documented at 48 hpi (the top panel) and 120 hpi (the middle and bottom panels). (**B**) Quantification of the lesion sizes caused by the indicated strains on the inoculated leaves as showing on (**A**). Data represent means ± standard deviations (SDs) from at least three independent experiments. ***: significance at P< 0.001.
