# Supplementary material for: Transcriptome analysis and functional validation reveal a novel gene, BcCGF1, that enhances fungal virulence by promoting infection‐related development and host penetration
Source: Mol Plant Pathol. 2020 Apr 16;21(6):834–53. doi: 10.1111/mpp.12934 (PMC7214349; doi:10.1111/mpp.12934)
Supplement: Supplementary file 6 — FIGURE S6 Loss of BcCGF1 reduces the virulence of Botrytis cinerea [file MPP-21-834-s006.docx]

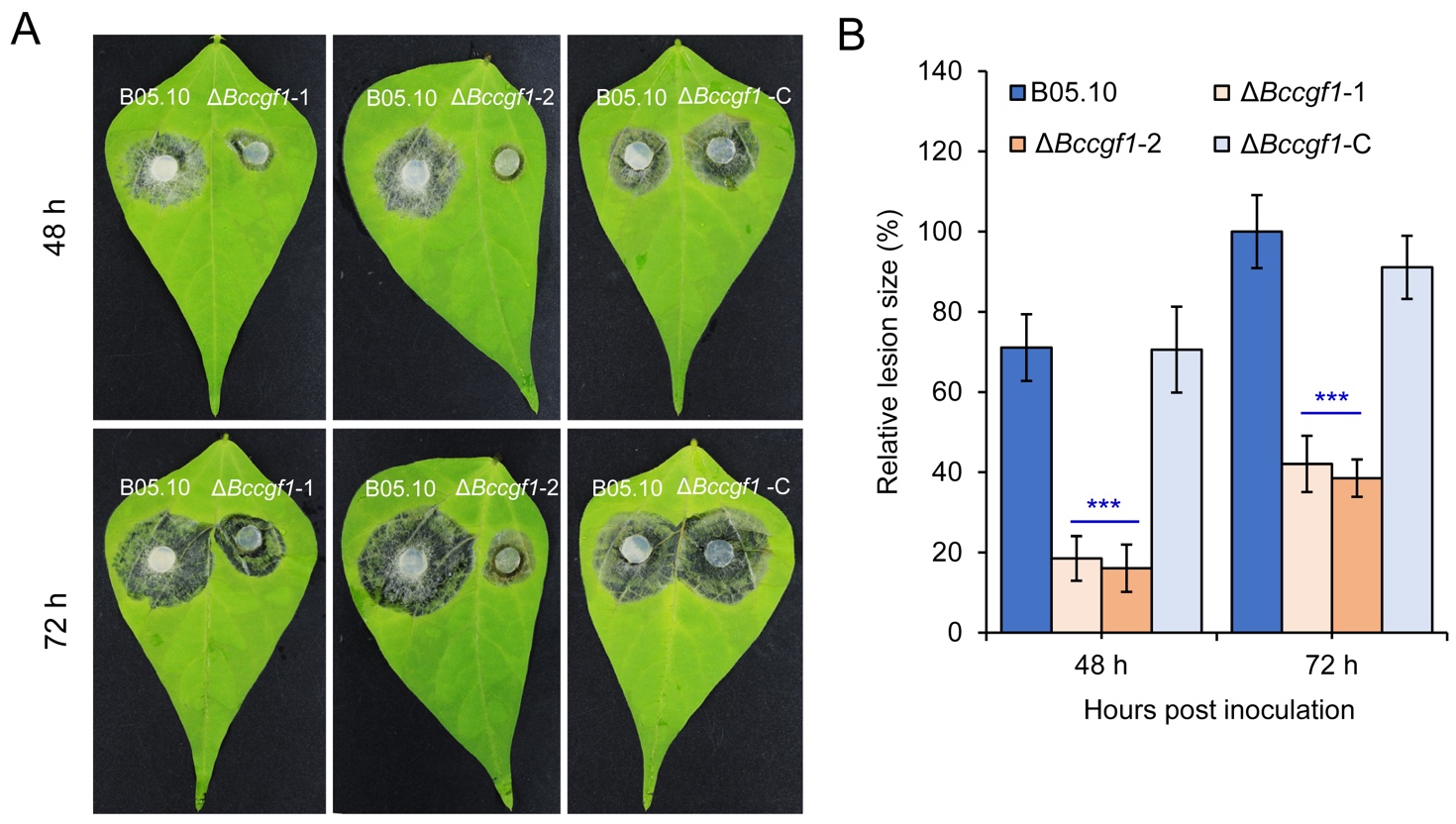


**Figure S6. Loss of *BcCGF1* reduces virulence of *B. cinerea.*** (**A**) Mycelial plugs of the B05.10, ∆*Bccgf1,* and ∆*Bccgf1-*C strains were inoculated on green bean leaves and the diseased leaves were photographically documented at 48 hpi and 72 hpi. (**B**) Quantification of the lesion sizes caused by the indicated strains on the inoculated green bean leaves at 48 and 72 hpi. Data represent means ± standard deviations (SDs) from three independent experiments. ***: significance at P< 0.001.
