# Supplementary material for: Transcriptome analysis and functional validation reveal a novel gene, BcCGF1, that enhances fungal virulence by promoting infection‐related development and host penetration
Source: Mol Plant Pathol. 2020 Apr 16;21(6):834–53. doi: 10.1111/mpp.12934 (PMC7214349; doi:10.1111/mpp.12934)
Supplement: Supplementary file 7 — FIGURE S7 BcCGF1 mediates endogenous reactive oxygen species production in Botrytis cinerea [file MPP-21-834-s007.docx]

**
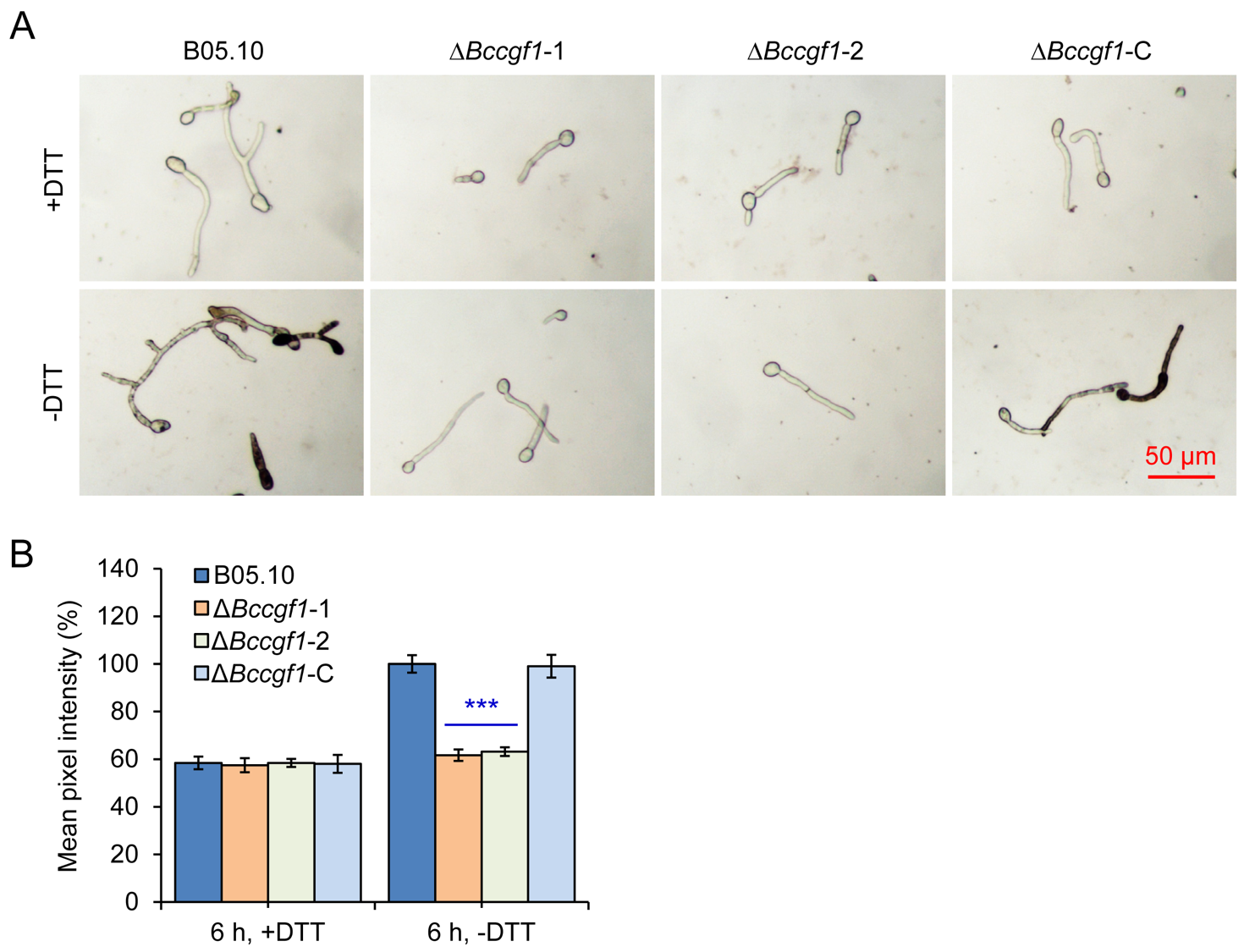
**

**Figure S7.** **Loss of *BcCGF1* in *B. cinerea* impairs the fungal endogenous ROS production.** Conidia of the WT, ∆*Bccgf1*, and ∆*Bccgf1*-C strains were cultivated on CM or CM supplemented with dithiothreitol (DTT) at 20°C for 6 h, and then the germ-tubes or hyphae of the tested strains were stained with DAB solution. (**A**) Disruption of *BcCGF1* reduced ROS production in the ∆*Bccgf1* mutants during appressorium formation detected by DAB staining. (**B**) Quantification of relative ROS production by the mean pixel intensity in the tested strains shown in (**A**). Data represent means ± SD from a representative experiment and three independent experiments were performed. ***: significance at p< 0.001.
