# Supplementary material for: Transcriptome analysis and functional validation reveal a novel gene, BcCGF1, that enhances fungal virulence by promoting infection‐related development and host penetration
Source: Mol Plant Pathol. 2020 Apr 16;21(6):834–53. doi: 10.1111/mpp.12934 (PMC7214349; doi:10.1111/mpp.12934)
Supplement: Supplementary file 8 — TABLE S1 Mapping results of RNA‐Seq reads [file MPP-21-834-s008.docx]

| Table S1. Mapping results of RNA-Seq reads | | | | |
| --- | --- | --- | --- | --- |
| **Sample Name** | **Total reads** | **Total mapped** | **Uniquely mapped** | **Multiple mapped** |
| SLL | 64518956 | 60503811(93.78%) | 5031933(0.78%) | 637989(0.99%) |
| B05.10 | 15754273 | 13100228(83.15%) | 13067511(82.95%) | 32171(0.21%) |
| B05.10_SLL vs SLL | 83128534 | 70186664(84.43%) | 69007583(83.01%) | 1179081(1.42%) |
| B05.10_SLL vs B05.10 | 83128534 | 5066784(6.10%) | 5044243(6.07%) | 22541(0.03%) |

SLL: Control of tomato

B05.10: Wild-type control of *Botrytis cinerea*

B05.10_SLL vs SLL: The mixed transcriptome mapped to tomato

B05.10_SLL vs B05.10: The mixed transcriptome mapped to the gray mold fungus
