# Supplementary material for: Transcriptome analysis and functional validation reveal a novel gene, BcCGF1, that enhances fungal virulence by promoting infection‐related development and host penetration
Source: Mol Plant Pathol. 2020 Apr 16;21(6):834–53. doi: 10.1111/mpp.12934 (PMC7214349; doi:10.1111/mpp.12934)
Supplement: Supplementary file 9 — TABLE S2 The percentages of the clean reads mapped to tomato and Botrytis cinerea genomes [file MPP-21-834-s009.docx]

Table S2. The percentages of the clean reads mapped to tomato and *B. cinerea* genomes

| **Sample** | **Raw reads** | **Clean reads** | **Clean bases** | **Error rate (%)** | Q20 (%) | **Q30 (%)** | **GC content (%)** |
| --- | --- | --- | --- | --- | --- | --- | --- |
| SLL | 33538774 | 32259478(96.2%) | 3.23G | 0.03 | 98 | 95.64 | 42.82 |
| B05.10 | 8273090 | 7877137(95.2%) | 0.79G | 0.04 | 97.26 | 92.07 | 46.67 |
| B05.10 vs SLL | 43717605 | 41564267(95.1%) | 4.16G | 0.03 | 97.01 | 91.97 | 42.85 |

SLL: Control of tomato (*S. lycopersicum cv*. Moneymaker)

B05.10: Wild-type control of *B. cinerea*

B05.10 vs SLL L: The mixed transcriptome of tomato and *B. cinerea*
