# Supplementary material for: Transcriptome analysis and functional validation reveal a novel gene, BcCGF1, that enhances fungal virulence by promoting infection‐related development and host penetration
Source: Mol Plant Pathol. 2020 Apr 16;21(6):834–53. doi: 10.1111/mpp.12934 (PMC7214349; doi:10.1111/mpp.12934)
Supplement: Supplementary file 21 — TABLE S14 Fungal strains used in this study [file MPP-21-834-s021.docx]

**Table S14. Fungal strains used in this study**

| Strain | Gene ID | Description | Origin |
| --- | --- | --- | --- |
| B05.10 |  | *B. cinerea* wild type strain | Lab collection |
| Δ*BcVEL1* | BCIN_15g03390 | *B. cinerea* *VEL1* Kockout mutant | This study |
| Δ*BcLAE1* | BCIN_05g01210 | *B. cinerea* *LAE1* Kockout mutant | This study |
| Δ*BcATG1* | BCIN_07g00720 | *B. cinerea* *ATG1* Kockout mutant | This study |
| Δ*BcCGF1* | BCIN_16g01820 | *B. cinerea* *CGF1* Kockout mutant | This study |
| Δ*BcCGF1-C* |  | Δ*BcCGF1* Complemented strain | This study |
| Δ*BCIN_03g01540* | BCIN_03g01540 | BCIN_03g01540 Kockout mutant | This study |
| Δ*BcBGL1* | BCIN_09g02640 | *B. cinerea* *BGL1* Kockout mutant | This study |
| Δ*BcBGL2* | BCIN_10g05590 | *B. cinerea* *BGL2* Kockout mutant | This study |
| Δ*BcBGL3* | BCIN_03g08710 | *B. cinerea* *BGL3* Kockout mutant | This study |
| Δ*BcBGL4* | BCIN_09g05460 | *B. cinerea* *BGL4* Kockout mutant | This study |
| Δ*BcBGL5* | BCIN_14g00650 | *B. cinerea* *BGL5* Kockout mutant | This study |
| Δ*BcBGL6* | BCIN_10g02650 | *B. cinerea* *BGL6* Kockout mutant | This study |
