# Supplementary material for: Transcriptome analysis and functional validation reveal a novel gene, BcCGF1, that enhances fungal virulence by promoting infection‐related development and host penetration
Source: Mol Plant Pathol. 2020 Apr 16;21(6):834–53. doi: 10.1111/mpp.12934 (PMC7214349; doi:10.1111/mpp.12934)
Supplement: Supplementary file 22 — TABLE S15 Primers used in this study [file MPP-21-834-s022.docx]

Table S15. Primers used in this study

| Primer name | Primer sequence（ 5’ to 3’） | Purposes |
| --- | --- | --- |
| CGF1UP1 | CTCGAGATCGTATTTATGATTGTGCCG | 5’-flank of *BcCGF1* for knock-out |
| CGF1UP2 | AGATCTATGATGATCGAAGTGGAGTGT | 5’-flank of *BcCGF1* for knock-out |
| CGF1D1 | GGATCCAAGGCTTCCAAGAACCAACG | 3’-flank of *BcCGF1* for knock-out |
| CGF1D2 | CTGCAGCCATAACATACCTCCCGCTG | 3’-flank of *BcCGF1* for knock-out |
| CGF1YZ1 | TTGGAGTGATTCCGACCGT | Diagnostic PCR for knock-out of *BcCGF1* |
| CGF1YZ2 | CGAACAGATGGATTCTTTATCAGTA | Diagnostic PCR for knock-out of *BcCGF1* |
| CGF1BM1 | TCAATATCTTGCTCGCTGTGG | Diagnostic PCR for knock-out of BcCGF1 |
| CGF1BM2 | GTTGGTTCTTGGAAGCCTTTC | Diagnostic PCR for knock-out of *BcCGF1* |
| CGF1HBCZ-1 | GATCTTCACTAGTGGGAATTCAGCGATGAGGAAAGGAAGAGC | Expression cassette of *BcCGF1* for complementation |
| CGF1HBCZ-2 | CCCGGTACCGAGCTCGAATTCGAGACGGAACGAACAGATGGA | Expression cassette of *BcCGF1* for complementation |
| CGF1RT1 | GAACAACGTGAGGCTCATCATACT | Quantitative real-time PCR |
| CGF1RT2 | ATTGTCCCTTGGATTTACCAGCT | Quantitative real-time PCR |
| HPTa-RC | ATGATGCAGCTTGGGCGCA | Diagnostic PCR for knock-out of mutant |
| HPTb-RC | ACAGACGTCGCGGTGAGTTCA | Diagnostic PCR for knock-out of mutant |
| HPTa | TGCGCCCAAGCTGCATCAT | Hygromycin internal primer |
| HPTb | TGAACTCACCGCGACGTCTGT | Hygromycin internal primer |
| LAE1UP1 | AGATCTCTATTTGCTCCCGTTGACTGT | 5’-flank of *BcLAE1* for knock-out |
| LAE1UP2 | GGTACCAAGCTTTTCTATGCGACCCTC | 5’-flank of *BcLAE1* for knock-out |
| LAE1D1 | GGATCC TCAAACCCCCTTCATAGACG | 3’-flank of *BcLAE1* for knock-out |
| LAE1D2 | CTGCAGGCCAGCACATTATTTTCCTC | 3’-flank of *BcLAE1* for knock-out |
| LAE1BM1 | CAGGTACCGGAATATGGGC | Diagnostic PCR for knock-out of *BcLAE1* |
| LAE1BM2 | GTCTGAGCGGGGAGTGAAA | Diagnostic PCR for knock-out of *BcLAE1* |
| LAE1YZ1 | GAATCAGGGTCGTTGTTTGCTA | Diagnostic PCR for knock-out of *BcLAE1* |
| LAE1YZ2 | CTAACCGTGTATCCAGTGTCCA | Diagnostic PCR for knock-out of *BcLAE1* |
| LAE1HB1 | CTGCAGCTCTCCTGCCTGCCCCGTAG | Expression cassette of *BcLAE1* for complementation |
| LAE1HB2 | GTCGACGGGTGCATTAAATCCCCAAC | Expression cassette of *BcLAE1* for complementation |
| VEL1UP1 | AGATCTTGGCGTGATTGAGAAGAGGGAT | 5’-flank of *BcVEL1* for knock-out |
| VEL1UP2 | GAATTCAGTTGAACGACGGGAGGAGGGT | 5’-flank of *BcVEL1* for knock-out |
| VEL1D1 | GGATCCTCTGTCGCTCTCGCACCACT | 3’-flank of *BcVEL1* for knock-out |
| VEL1D2 | CTGCAGATTCCGTTCCGTTCCATTCC | 3’-flank of *BcVEL1* for knock-out |
| VEL1BM1 | TCGCAACTTTGGAGGTAGC | Diagnostic PCR for knock-out of *BcVEL1* |
| VEL1BM2 | GAGGGGTTTGTGGGAGGAC | Diagnostic PCR for knock-out of *BcVEL1* |
| VEL1YZ1 | GTGTGTTGCTGACTGCCCA | Diagnostic PCR for knock-out of *BcVEL1* |
| VEL1YZ2 | ATGGAACCAACTCATCTGCG | Diagnostic PCR for knock-out of *BcVEL1* |
| VEL1HB1 | CTGCAGTAGTCCGTCAACCAGTCATTT | Expression cassette of *BcVEL1* for complementation |
| VEL1HB2 | GAATTCTCTGCTTGTTTTCTCTCCATT | Expression cassette of *BcVEL1* for complementation |
| ATG1UP1 | CTCGAGAATACGCTTCTTTCTTCTTTC | 5’-flank of *BcATG1* for knock-out |
| ATG1UP2 | GAATTCAATATCCTTCTCAGTTCCTTG | 5’-flank of *BcATG1* for knock-out |
| ATG1DN1 | GGATCCTCGGGCGTCATTCGAGTTTA | 3’-flank of *BcATG1* for knock-out |
| ATG1DN2 | CTGCAGTGAGTGGTGGAGCGGGTGGT | 3’-flank of *BcATG1* for knock-out |
| ATG1BM1 | TAAGAACTGGGTCCACGAAAC | Diagnostic PCR for knock-out of *BcATG1* |
| ATG1BM2 | TCAGCAAATCCATAAACAACA | Diagnostic PCR for knock-out of *BcATG1* |
| ATG1YZ1 | ACATCTTCCTGCTCCGACAT | Diagnostic PCR for knock-out of *BcATG1* |
| ATG1YZ2 | TTTGGAAAGGAGGAGGTTGC | Diagnostic PCR for knock-out of *BcATG1* |
| ATG1HBCZ-1 | GATCTTCACTAGTGGGAATTCCTTCACTCTTCACTACCAGCCTTCA | Expression cassette of *BcATG1* for complementation |
| ATG1HBCZ-2 | CCCGGTACCGAGCTCGAATTCCAAATCCCACCCAAACACCAA | Expression cassette of *BcATG1* for complementation |
| BCIN_03g01540UP1 | GAATTCTGCGGTGTATTCTGTGGTT | 5’-flank of *BCIN_03g01540* for knock-out |
| BCIN_03g01540UP2 | GGTACCTGCAAAGGTGGAAAGTAGG | 5’-flank of *BCIN_03g01540* for knock-out |
| BCIN_03g01540D1 | GGATCCCATCGCTCCTCACCTTCAT | 3’-flank of *BCIN_03g01540* for knock-out |
| BCIN_03g01540D2 | AAGCTTTCAAAATCTCCATAGTCCG | 3’-flank of *BCIN_03g01540* for knock-out |
| BCIN_03g01540BM1 | GACTTTGGGAGGTTGTTCTG | Diagnostic PCR for knock-out of BCIN_03g01540 |
| BCIN_03g01540BM2 | CGGAGGTGATGGTAAGGGAT | Diagnostic PCR for knock-out of B BCIN_03g01540 |
| BCIN_03g01540YZ1 | GCCTAAGGCACGGATCTAAT | Diagnostic PCR for knock-out of BCIN_03g01540 |
| BCIN_03g01540YZ2 | AAGAGTGGAAACTGCGGGTAG | Diagnostic PCR for knock-out of BCIN_03g01540 |
| BGS1Up1 | CTCGAGAGGCAAGGATACTTACCACC | 5’-flank of *BcBGL1* for knock-out |
| BGL1Up2 | AGATCTGTAAATCTTCGGGAACTCAT | 5’-flank of *BcBGL1* for knock-out |
| BGL1Down1 | GGATCCACTTTGGGAGGAATGGTGAG | 3’-flank of *BcBGL1* for knock-out |
| BGL1Down2 | CTGCAGCTTGCTTCGTGGATGACTGC | 3’-flank of *BcBGL1* for knock-out |
| BGL1BM1 | GCTGGAAGAAATTGGGAAGG | Diagnostic PCR for knock-out of *BcBGL1* |
| BGL1BM2 | GAGGGAGTGCGTTGTTGGTAT | Diagnostic PCR for knock-out of *BcBGL1* |
| BGL1YZ1 | GGCGGTGACCTGAGTTCTG | Diagnostic PCR for knock-out of *BcBGL1* |
| BGL1YZ2 | ATTTGGTTTGGCTGGTGAG | Diagnostic PCR for knock-out of *BcBGL1* |
| BGL1RT1 | GCAGCGAAACACTGAAGGAA | Quantitative real-time PCR |
| BGL1RT2 | TTTGACAGGCGTAGGATGAG | Quantitative real-time PCR |
| BGL2Up1 | CTCGAGCCACTCGACCAGGGAACTAG | 5’-flank of *BcBGL2* for knock-out |
| BGL2Up2 | GAATTCGGCGGTAATGAAGAAGAACG | 5’-flank of *BcBGL2* for knock-out |
| BGL2Down1 | GGATCCAACCCAGCACTTTACGACGTG | 3’-flank of *BcBGL2* for knock-out |
| BGL2Down2 | AAGCTTGCATCTCACAACCAGCATAGC | 3’-flank of *BcBGL2* for knock-out |
| BGL2BM1 | CAATCTGGCGAATCACAAGGC | Diagnostic PCR for knock-out of *BcBGL2* |
| BGL2BM2 | GGTAAGGGAAGTTGGCGGAAC | Diagnostic PCR for knock-out of *BcBGL2* |
| BGL2YZ1 | TTCCCTTCTTTCGCCCAACT | Diagnostic PCR for knock-out of *BcBGL2* |
| BGL2YZ2 | ACACTCAACACGAAAGAAAC | Diagnostic PCR for knock-out of *BcBGL2* |
| BGL2RT1 | TGGTCGTGCTCCACAAGGTG | Quantitative real-time PCR |
| BGL2RT2 | CCGTGTATTGATAATCGGAAAGTA | Quantitative real-time PCR |
| BGL3Up1 | CTCGAGGTACGACGCATGACTTAGGGA | 5’-flank of *BcBGL3* for knock-out |
| BGL3Up2 | GAATTCGAGAAAAGAGGTGGGGATTTA | 5’-flank of *BcBGL3* for knock-out |
| BGL3Down1 | GGATCCAGATTGTTGGTATGCAGGAC | 3’-flank of *BcBGL3* for knock-out |
| BGL3Down1 | CTGCAGGGCGGTGAATATGAAGTTGT | 3’-flank of *BcBGL3* for knock-out |
| BGL3BM1 | AAATACTGGCACAATCCCTCG | Diagnostic PCR for knock-out of *BcBGL3* |
| BGL3BM2 | GCCCACCAATCACTCACAACT | Diagnostic PCR for knock-out of *BcBGL3* |
| BGL3YZ1 | GTCATCTTACTACCCGCCTCC | Diagnostic PCR for knock-out of *BcBGL3* |
| BGL3YZ2 | CGATGGCTCAACGAATGTC | Diagnostic PCR for knock-out of *BcBGL3* |
| BGL3RT1 | AATACTGGCACAATCCCTCG | Quantitative real-time PCR |
| BGL3RT2 | CTGGCCCATAGCAACACCTC | Quantitative real-time PCR |
| ACT-F | CATGGCTGGTCGTGATTTGA | Quantitative real-time PCR |
| ACT-R | GAGGATTGACTGGCGGTTTG | Quantitative real-time PCR |
| BGL4Up1 | CTCGAGTGCACGGTTACATACAGACG | 5’-flank of *BcBGL4* for knock-out |
| BGL4Up2 | AGATCTATTGGTTGACGAAGGTGAGA | 5’-flank of *BcBGL4* for knock-out |
| BGL4Down1 | GGATCCTGGAGAAATGAATGAGGGAG | 3’-flank of *BcBGL4* for knock-out |
| BGL4Down2 | CTGCAGTCGTGGGTGAAATACTTGGT | 3’-flank of *BcBGL4* for knock-out |
| BGL4BM1 | GGCAAATATGTACGGCTATGA | Diagnostic PCR for knock-out of *BcBGL4* |
| BGL4BM2 | ATCGTCATTACCCATTCATCC | Diagnostic PCR for knock-out of *BcBGL4* |
| BGL5Up1 | CTCGAGGCTACATAGACTTCCCTCATACA | 5’-flank of *BcBGL5* for knock-out |
| BGL5Up2 | AGATCTATGGGATTGACGATATTGACT | 5’-flank of *BcBGL5* for knock-out |
| BGL5Down1 | GTCGACCTGGAGAAATAGTTCTTAGCGTAC | 3’-flank of *BcBGL5* for knock-out |
| BGL5Down2 | CTGCAGAGTTGCGTCTGGAATGGTGG | 3’-flank of *BcBGL5* for knock-out |
| BGL5BM1 | TTACACGAGCAAAGACAGGC | Diagnostic PCR for knock-out of *BcBGL5* |
| BGL5BM2 | AATCCTCATGGTCGATCCTG | Diagnostic PCR for knock-out of *BcBGL5* |
| BGL6Up1 | CTCGAGATGCTACAAGTCCAGGTGAA | 5’-flank of *BcBGL6* for knock-out |
| BGL6Up2 | AGATCTAATCAGTTGTTGCGAGAAGG | 5’-flank of *BcBGL6* for knock-out |
| BGL6Down1 | GGATCCGGGTTTACTCAGTGGGTGGTT | 3’-flank of *BcBGL6* for knock-out |
| BGL6Down2 | CTGCAGTCGGCAATTTGGTACAGCAT | 3’-flank of *BcBGL6* for knock-out |
| BGL6BM1 | CCCGAAAGTTAAGAATTGGAT | Diagnostic PCR for knock-out of *BcBGL6* |
| BGL6BM2 | CTCACCTATTCCCTCCCTTTC | Diagnostic PCR for knock-out of *BcBGL6* |
